# Valorization of Anaerobic-Fermentation Liquid Digestates—Membrane-Based Process Development

**DOI:** 10.3390/membranes13030297

**Published:** 2023-03-01

**Authors:** Charikleia Tsaridou, Anthoula Karanasiou, Konstantinos V. Plakas, Anastasios J. Karabelas

**Affiliations:** Laboratory of Natural Resources and Renewable Energies, Chemical Process and Energy Resources Institute, Centre for Research and Technology-Hellas (CERTH), 57001 Thermi-Thessaloniki, Greece

**Keywords:** anaerobic fermentation, liquid digestate valorization, membrane process treatment, P, N nutrients, water recycling

## Abstract

Complete valorization of various wastes and effluents, with significant organic content, remains a great challenge in the pursuit of a circular economy. The approach based on anaerobic fermentation, leading to valuable biogas production, has been broadly accepted and employed as an attractive processing scheme. However, despite notable research efforts, complete valorization of the digestates (involving recovery of nutrients/by-products and full recycling/reuse of treated water) requires additional work for sustainable process development. This study aims to make a contribution in this direction by demonstrating a systematic methodology for valorizing the liquid digestate. The proposed membrane-based processing scheme involves UF-membrane pretreatment of the liquid digestate (for sludge separation) and subsequent NF/RO membrane treatment for reuse/recycling of the permeate; the concentrate, enriched in “nutrients” (phosphate and ammonium compounds), can be utilized for soil fertilization, with further conditioning/processing. By performing targeted laboratory experiments and advanced simulations, the membrane-based process was developed to a relatively high technology-readiness level, including a pilot unit design/construction and preliminary testing with satisfactory results. Through pilot testing in industrial environment, further process development and optimization will be pursued, towards practical applications. The demonstrated methodology is also considered appropriate for systematic development of membrane-based processes to valorize/treat a variety of similar effluents.

## 1. Introduction—Scope

The unexploited potential of various effluents/wastes, combined with environmental regulations and related initiatives [1,2], have intensified activities toward development of integrated process systems, aiming to valorize them, particularly in the food- and agro-industry sectors [3,4]. In parallel, the worldwide repletion and degradation of clean water resources [5], along with the foreseen scarcity of “macronutrients” (mainly compounds of phosphorus and nitrogen, contained in such effluents) [6], which are essential for crop production and animal husbandry, have directed attention to the development of strategies ensuring circularity in agro-food-waste systems [4].

Anaerobic digestion (AD) is an established process for the valorization of organic wastes through biogas production [5,7], and it has good prospects (after enhanced treatment of effluents/digestates) for production of valuable biochemical products [3,5,8]. Approximately 19,000 such biogas plants operate in Europe (2020) [9], with more than 80% of the waste, fed into the AD unit, ending up as digestate, i.e., the raw mixed liquor of the AD process [10]. Usually, the suspended solids in the mixed liquor (10–20% by mass) are initially separated/filtered in the form of sludge [11]. Sludge processing and utilization is fairly common (e.g., [12,13]) and will not be considered here. The remaining large volume of liquid (for further treatment) will be referred to here as “filtered liquid digestate” (FLD). The significant nutrient content of FLD [13,14] render its valorization a matter of great importance [15]. However, sustainable valorization of FLD, considering its characteristics (i.e., salinity, pH, nutrients, and other possible organic/inorganic micropollutants), is rather complicated [16]. Indeed, particular processing is necessary to recover useful nutrients and obtain treated water (final effluent) preferably appropriate for reuse/recycling or safe disposal [17], thus avoiding secondary pollution [13,18,19]. Significant R&D efforts have been made for testing/application of various treatment processes, including chemical precipitation, air and steam stripping [20,21,22,23,24,25], evaporation, ion exchange, adsorption [25], and membrane processes [23,26,27]. The efficiency, technology readiness level, and economic feasibility of these processes are discussed in several recent reviews [7,15,17,19,28,29], which indicate that significant R&D work lies ahead to develop such sustainable processes for various types of digestates.

Some typical processes investigated so far, and related issues faced, are briefly outlined here. Precipitation of useful salts (calcium phosphate and struvite) is a fairly well tested option for recovery of nutrients; however, technical/economic issues such as consumption of chemicals, end-product impurities and process instability seem to hinder wide application of this process type [15,17]. Ammonia (NH_3_) is usually recovered through air/steam stripping and sulfuric acid scrubbing in the form of ammonium sulfate [17,30]. This product, although valuable, is reportedly confronted with farmers’ mistrust regarding its fertilizing attributes [15,31]. Ion exchange and adsorption are considered rather simple and low-cost separation techniques [17,32,33], but they must be applied to digestate with low suspended solids (SS) content, which necessitates FLD pretreatment to avoid clogging of the packed column [34]; this is apparently the reason the process has not been demonstrated at full scale yet. Nevertheless, it may be integrated with other processes, e.g., coupling of zeolite adsorption with struvite precipitation [15] for production of fertilizers. 

Membrane processes tested for FLD treatment include microfiltration (MF), ultrafiltration (UF), forward osmosis (FO), membrane distillation (MD), electrodialysis (ED), nanofiltration (NF), and reverse osmosis (RO) [5,26,34,35]. MF and UF are commonly used for the pretreatment of digestate, to remove SS and macromolecules [34], whereas NF and/or RO are employed for selective separation of inorganic ions, including ammonium (NH_4_^+^) and ortho-phosphate (PO_4_^3−^) ions [13,36]. Despite the well-known attributes of membrane technology and related useful research [18,26,37,38,39], there are still significant issues (including optimal membrane selection and design, membrane fouling, and scaling [39,40]) that hinder industrial applications. Typical relevant R&D activity is briefly outlined next.

Gerardo et al. [37] at laboratory scale and Zacharof et al. [18] in a pilot investigated the valorization of anaerobic digestates, from treatment of livestock and agricultural wastes, focusing on the fractionation of nitrogen and phosphorus compounds (i.e., ammonia/ammonium and phosphate ions, respectively) in separate product streams. Aiming to maximize effective nutrient separation, the liquid digestates were significantly diluted with water and then treated with specific membrane types (i.e., NF [37] and sequential UF-NF [18]), employing the diafiltration (DF) mode. The feasibility of fractionating the N, P nutrients (under appropriate pH) was demonstrated [18,37], although no attention was paid to overall management or reuse of the treated effluents. Fernandes et al. [39] studied the valorization of digestate from kitchen/food waste at laboratory scale, using dilution, settling, and a combination of MF, UF, and NF membranes. Significant recovery (~95%) of both N and P was obtained as well as reduction of color and heavy metals in the NF permeate, which was considered potentially dischargeable to the environment. Moreover, MF and NF permeates at rather small nutrient concentrations, were shown at pilot scale to be suitable substrates for successful microalgal cultivation. Magalhães et al. [26] in laboratory investigation integrated NF and RO to UF and a two-stage anaerobic membrane bioreactor (treating sugarcane vinasse) recovering nutrients and water from NF/RO concentrate and permeate, respectively; they concluded that the RO permeate could be recycled for industrial uses. Adam et al. [38] evaluated a sequential NF and two-stage RO membrane process (for separating organic matter, nutrients, and water from digestate), in pilot units installed at two biogas plants processing agricultural, food-industry, and livestock waste. Two groups of “fertilizers” were produced from the digestate solid fraction and NF retentate; however, process improvement was deemed necessary regarding the quality of permeate (i.e., reduction of NH_4_^+^ and K concentration) to allow safe discharge. It was concluded that achieving a good-quality final effluent critically affects the design of the entire process scheme.

From the preceding overview, it is concluded that, although anaerobic digestion (for biogas production) is a widely implemented process at large/industrial scale, further R&D efforts are needed, regarding the treatment of the (polluting) significant volumes of FLD, aiming at both (*a*) recovery/reuse of contained nutrients (mainly P and N compounds) and (*b*) removal of pollutants from the final effluents for recycling or safe disposal. Indeed, although useful results have been obtained, no established integrated processing schemes are available (to the authors’ best knowledge) for complete FLD valorization. This study aims to contribute toward this direction, by demonstrating a systematic methodology, involving targeted experiments and comprehensive computer simulations for development of a membrane-based process. The methodology is outlined first, followed by implementation for an industrial digestate.

## 2. Methodology

### 2.1. General Considerations—Effluent Treatment Scheme, Tasks

FLD is generally characterized by seasonal variability and a significant level of Total Dissolved Solids (TDS), organic load, and possibly other pollutants (e.g., heavy metals), depending on the composition of feed to the fermentor [13,19]. Considering the relevant in-effect EU and other regulations [2,41], such waste streams cannot be reused or safely disposed (as shown in Table A1 of Appendix A), unless properly treated. 

**Figure 1:**
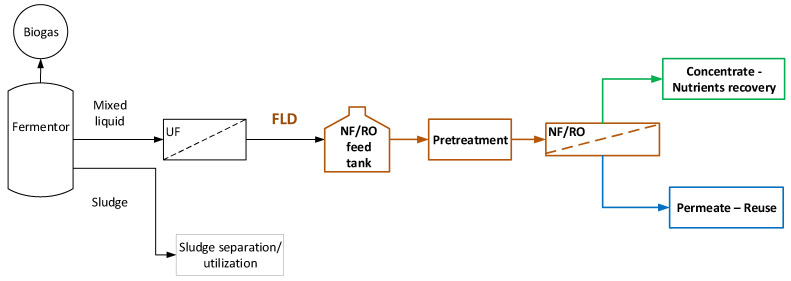
Block flow diagram of *membrane-based* process for *complete valorization* of anaerobic fermentation liquid digestate (FLD).

The general processing scheme, depicted in Figure 1, is considered in this study to serve the objective of *complete FLD valorization*; i.e., nutrients’ recovery and treated effluent suitable for full reuse/recycling. Typical FLD is obtained from an industrial plant, installed and operating in a dairy industry in Central Greece (BIZIOS S.A.). The plant is comprised of an anaerobic fermentor (producing biogas), an excess-sludge separation system and a UF unit (Memthane^®^ [42]) leading to FLD free of solids. Further main processing steps, examined here, include (i) *FLD pretreatment* and (ii) an *NF/RO membrane separation unit* targeted to yield a permeate appropriate for reuse/recycling and a concentrate stream to be utilized for nutrient recovery/reuse, after possible conditioning/processing.

In summary, the basic issues and tasks, addressed in the ensuing membrane-based process development, include: (a) FLD pretreatment type; (b) selection of appropriate membrane type, and determination of a relatively narrow range of near optimal process conditions (to achieve the project targets); (c) means/procedures for dealing with membrane fouling, scaling, and cleaning; and (d) designing, building, and preliminary testing of an appropriate industrial membrane pilot, necessary for further process development. Additional future tasks to bring the process development to a high technology readiness level (TRL~7/8) include (e) operating the membrane pilot unit in the industrial site and collecting necessary process performance data; (f) examining potential options for nutrients utilization from NF/RO concentrate, e.g., recovery in the form of struvite [43,44]); and (g) optimization and sustainability assessment of the entire processing scheme. This paper reports on tasks (a) to (d), whereas the ensuing tasks (e, f, g) are in progress.

### 2.2. Outline of Methodology

The sequential steps comprising the proposed methodology are summarized as follows:

I. *Bench scale testing—Dead-end membrane tests*

Screening of candidate membrane types and preliminary assessment of their performance is carried out, based on main process parameters; i.e., membrane–material compatibility, species rejection characteristics, and effective membrane permeability. Results to be obtained include: selection of few appropriate membrane types for further testing.

II. *Laboratory membrane process performance assessment—Cross-flow equipment tests*

Such experimental set-up (including test sections with spacer-filled membrane channels) is realistic and representative of conditions in industrial membrane modules, thus allowing:

- General membrane/process performance assessment and selection of best membrane type for further comprehensive testing.
- Comprehensive membrane process performance testing. Adequate data collection for key parameters and assessment of main effects, i.e., membrane-effective permeability, species rejection/separation, membrane fouling characteristics, and membrane cleaning.

III. *Process performance simulations—Pilot plant design*

Using appropriate software, detailed simulation is performed of a realistic process, i.e., design of a unit involving pressure vessel(s), with commercial spiral wound membrane (SWM) modules of the selected membrane type, aiming at:

- Validation using comparison of simulation results with experimental process performance data from preceding step II.
- Identification of a (narrow) range of near-optimum design and operating process conditions.
- Basic design data for an appropriate industrial membrane pilot, based on the foregoing results.

IV. *Pilot plant construction and preliminary testing*

Further steps, to be dealt with in future papers include:

V. *Industrial pilot testing*

- Performance data collection, to validate the methodology and select key process parameters.
- Refinement of projections of a real plant performance, aiming to finalize process design.

VI. *Process optimization, techno-economic/sustainability assessment*

## 3. Experimental Design—Equipment, Materials, Techniques

### 3.1. Materials, Techniques

Preliminary screening tests were performed with synthetic solutions and commercial flat sheet Ultra Low Pressure RO (ULPRO) and NF membranes, i.e., NF90, XLE (DuPont-FilmTec™, Edina, MN, USA), TS80, and ACM2 (MICRODYN-NADIR GmbH, Wiesbaden, Germany). The specifications of the pre-selected membranes (Table S1, Supplement) seem to serve the target of the study (effective recovery of nutrients and permeate/water of fair quality for reuse) due to reported high monovalent and divalent ion rejection (~90 and 99%, respectively). Furthermore, all four membranes are tolerant of modest fluid temperatures (up to ~35 °C) and exhibit good performance in the presence of various inorganic and organic compounds [45,46,47,48].

Representative synthetic solutions (Table A2, Appendix A) were prepared by dissolving, in deionized (DI) water, ammonium chloride (NH_4_Cl) and potassium dihydrogen phosphate (KH_2_PO_4_), purchased from Lachner S.R.O. (Slovakia) and ΡΕΝΤA S.R.O. (Czech Republic), respectively. The ion concentration of NH_4_-N and PO_4_-P (~150 mg/L each) was close to that of the FLD (Table A1, Appendix A) obtained from the aforementioned operating anaerobic fermentation unit after filtration of the raw fermentation digestate (Figure 1), using Pentair X-Flow hollow fiber UF membranes [42] of MWCO 150–200 kDa.

Detailed physicochemical characterization of various process solutions was conducted with the instrumentation presented in Table S2 (Supplement). Scanning Electron Microscopy (SEM) and Energy-Dispersive Spectroscopy (EDS) were used to assess membrane condition and deposits before and after the FLD filtration (Table S2, Supplement). An estimate of the foulants’ density on the membrane surface was also obtained via a dissolution technique [40]. The latter involved immersion and agitation of membrane samples (of measured surface area) in solutions of HCl 60 mM and of NaOH 25 mM, for the respective inorganic and organic foulants’ dissolution and subsequent determination. 

The tendency of tested aqueous solutions for solid-phase formation [49] and membrane scaling was quantified using a key parameter, the supersaturation ratio, defined as:

(1) $$S={\left[\frac{{\left(M^{a+}\right)}^{n}{\left(A^{b-}\right)}^{m}}{K_{sp}}\right]}^{1/\left(n+m\right)}={\left[\frac{IAP}{K_{SP}}\right]}^{\frac{1}{n+m}}$$

where:

- *M^α+^* and *A^b−^* are the activities of the ionic species of a compound M_n_A_m_.
- *K_sp_* is the thermodynamic solubility product of the solid forming compound.
- *IAP* is the respective ion activity product.

The Supersaturation Index (*SI*), where *SI* > 0 designates supersaturation, was also used:

(2) $$SI=\frac{1}{n+m}\log_{10}\left(\frac{IAP}{K_{sp}}\right)$$

where *SI* is related to *S* as follows:

(3) $$SI=\left(n+m\right)\log S$$

The supersaturation ratio *S* and saturation index *SI* of process solutions were determined through a special software, PHREEQC (version 3.6.2.15100) [50]. PHREEQC is based on a thermodynamic ion-association model implementing the extension of the Debye–Hückel theory. For solutions with relatively low salinity (as for FLD), the “minteq.v4.dat” database was employed [51]. 

A parameter necessary to assess the membrane performance is the (potentially varying) effective permeability, *K_p_*, determined by the measured permeate flux, *J*, and the effective transmembrane pressure (Δ*P* − Δπ), at a certain time or % recovery, as follows:

(4) $$K_{p}=\frac{J}{\Delta Ρ-\Delta \pi }$$

The effective osmotic pressure difference Δπ is calculated considering the species concentration at the membrane surface due to concentration polarization phenomena [40]:

(5) $$\frac{C_{w,\;i}}{C_{b,i}}=\left(1-R_{i}\right)+R_{i}\cdot \exp\left(\frac{J}{k_{i}}\right)$$

where:

- *C_w,I_*, *C_b,i_*: Concentration of ions at the membrane surface and in bulk solution, respectively.
- *R_i_*, *J*: Measured ions’ rejection and permeate flux, respectively.
- *k_i_*: Mass transfer coefficient at the membrane surface determined by a reliable correlation [52].

### 3.2. Bench—Scale Equipment, Procedures—Dead-End Cells

The screening of candidate membrane types and preliminary assessment of their performance was executed in dead-end mode under constant agitation. The experimental set-up (Figure S1, Supplement) comprised a pair of high pressure thermostated stirred cells (SEPA-ST cells, Osmonics Inc., Minnetonka, MN, USA) [53], each with maximum capacity 0.3 L, inner diameter 4.7 cm, and effective membrane area 12.7 cm^2^. A constant/controlled feed pressure of 5 bar was provided using a nitrogen gas cylinder, and the stirring rate was maintained at 250 rpm; the latter results in an average shear stress at the membrane surface, close to that usually prevailing in spacer-filled channels of SWM modules [54]. A cooling system (PolyScience, 9106, Niles, IL, USA) ensured temperature stabilization at 25 ± 0.2 °C. Each filtration test was performed with a new/fresh membrane specimen and the permeate flux was determined using monitoring permeate mass.

### 3.3. Cross-Flow Equipment, Procedures

This part of the study involved membrane/process performance tests, in a laboratory cross-flow filtration set-up, under conditions considered representative of those prevailing *locally* in real SWM elements. These experiments were also conducted under constant pressure, at 25 °C, cross-flow velocity ~20 cm/s, mainly in batch mode, i.e., with full recycling of concentrate. However, once-through flow tests, under constant feed pressure and concentration (i.e., recycling of both permeate and concentrate) were also possible. The merits of experimenting with each mode are discussed in following sections.

**Figure 2:**
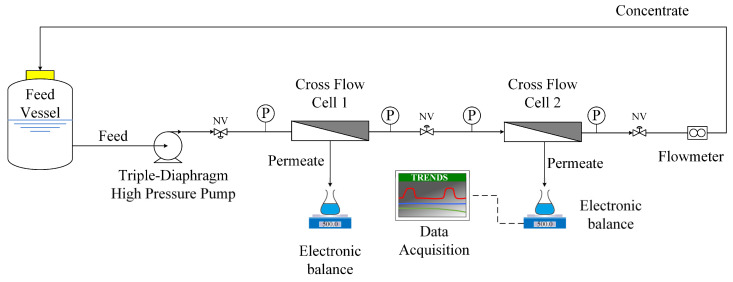
Experimental set-up employed in membrane filtration tests, in the cross-flow mode.

The employed experimental set-up, depicted in Figure 2, has been described in previous work [55]. The unit included two test sections of narrow (1 mm) gap, employing flat sheet membrane pieces of filtration area 130.35 cm^2^ (5.5 cm × 23.7 cm) as well as a common commercial net-type spacer. The feed solution was recirculated via a triple-diaphragm high-pressure pump (P200MSTSSA05A, Wanner Intern. Ltd., UK), and the permeate mass rate was continuously recorded with an electronic balance (PL602-S, Mettler-Toledo, Columbus, OH, USA) connected to a computer for automatic data acquisition (GeniDAQ, Advantech Co. Ltd., Taipei, Taiwan). A substantial amount of original feed fluid was employed (~6–8 L, in a temperature stabilized feed vessel) to enable smooth experimentation for long-time tests. Samples of all process streams could be collected periodically. Pictures of this experimental set-up are included in the Supplemental Materials (Figure S2).

## 4. Methodology Implementation—Results

### 4.1. Dead-End Tests

**Figure 3:**
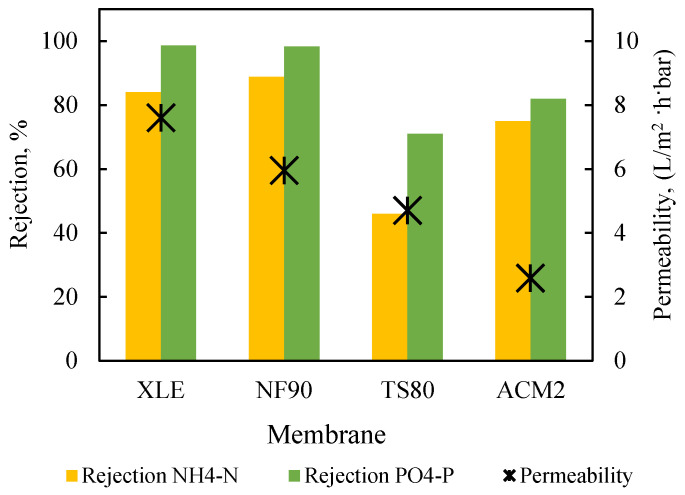
Performance of XLE, NF90, TS80, and ACM2 membranes regarding permeability and nutrient rejection; constant pressure 5 bar. Feed: synthetic solutions.

The four candidate membranes were assessed with the determination of (a) pure water permeability (at applied pressures of 2, 4, 6, and 8 bar) and (b) simultaneous rejection of NH_4_-N and PO_4_-P from synthetic solutions (alternatively termed “binary”) presented in Table A2, at constant applied pressure of 5 bar and test duration of 1 h. Prior to all filtration tests, the membranes were compacted at 10 bar for 1h, without agitation. The results depicted in Figure 3 show that the two DuPont membranes exhibited the highest clean water permeability (XLE, 7.6 L/m^2^·h·bar; NF90, 6.6 L/m^2^·h·bar) and the highest rejection of nutrients (i.e., [NH_4_-N 84% and 89%] and [PO_4_-P 99% and 98%], for XLE and NF90, respectively) in accord with the manufacturer’s specifications for NaCl solutions [45,46]. Permeate flux *J* remained substantially constant throughout these tests, due to the filtration of relatively dilute solutions, while the highest value (32.6 L/m^2^·h) was obtained with the XLE membrane (compared to 29.7 L/m^2^·h for NF90), as shown in Table S3 of the Supplemental Materials.

Nutrient rejection by NF90 and XLE membranes was not affected when experiments were performed with solutions of single ammonium or phosphate ions (Table S3, Supplement), indicating the rather negligible effect of the coexisting ions and concentration polarization. 

The increase of the operating pressure (i.e., 3, 5, and 7 bar) resulted, as expected, in higher fluxes, for both XLE and NF90 membranes (Figure S3), but it had insignificant effect on rejection, indicating that these membranes would be appropriate for applications in a relatively wide range of operating conditions. 

**Figure 4:**
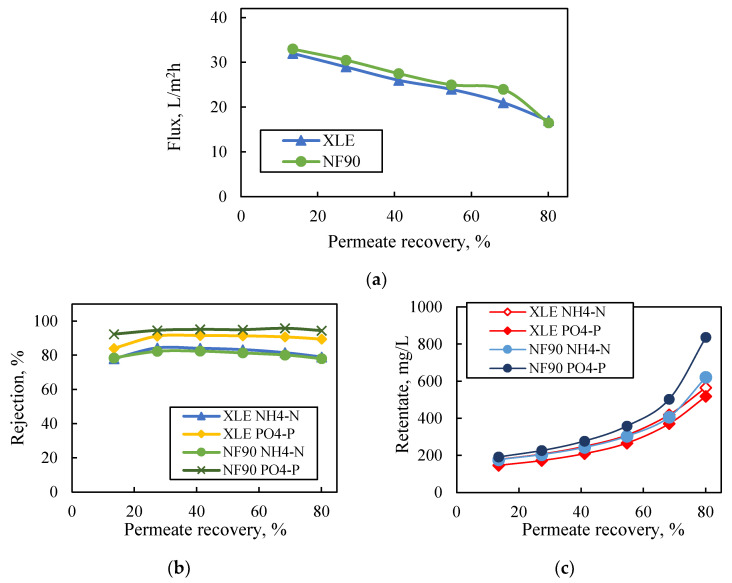
Effect of permeate recovery on (**a**) flux, (**b**) nutrient rejection, (**c**) nutrient concentration in retentate. Feed: synthetic solutions of NH4-N and PO4-P (150 mg/L each); NF90 and XLE membranes. Dead-end filtration, constant pressure 5 bar.

The performance of NF90 and XLE membranes at high permeate-recovery rates (ca. 80%) under constant agitation (250 rpm) and constant pressure (5 bar) is presented in Figure 4. Permeate flux exhibited progressive reduction (similar for both membranes) reaching ~50% of the initial value (i.e., from 31.5 L/m^2^·h to 14.5 L/m^2^·h and 33 L/m^2^·h to 16.5 L/m^2^·h for XLE and NF90, respectively), due to the increase of solution osmotic pressure. The rejection of nutrient ions was constantly high (PO_4_-P > 90% and NH_4_-N > 80%), even at 80% permeate recovery, with the highest rejection values obtained for P-PO_4_ ions by NF90 (ca. 95%). The consistently high rejection of NF and ULPRO membranes was also observed by Magalhães et al. [26], who investigated the treatment of the permeate of a two-stage anaerobic membrane bioreactor using NF90 and BW30 (DuPont-FilmTec™, the latter similar to XLE). The final retentate was 5 times and 4 times concentrated regarding PO_4_^3−^ ions by NF90 and XLE, respectively; NH_4_^+^ ions were concentrated 3.9 and 3.6 times, with NF90 and XLE, respectively.

In summary, the results from dead-end tests with synthetic solutions suggest that the NF90 and XLE membranes, in general, perform well and are considered appropriate for the intended task. Moreover, the obtained significant concentration of the two nutrient ions in the reject stream of both membranes (desirable for nutrient recovery or potential utilization of this concentrate in liquid fertilizer) warrants further investigation of NF90 and XLE membrane performance, under more representative membrane process conditions, as follows.

### 4.2. Cross-Flow Tests

#### 4.2.1. Assessment of Membrane Fouling/Scaling Propensity and FLD Pretreatment

##### Permeability and Rejection Tests with Synthetic Solutions

NF90 and XLE were tested in cross-flow mode using synthetic solutions with permeate recovery reaching 75% (Table S4). In these tests, the (comparatively more permeable) NF90 membrane displayed in general quite good performance, with initial and final flux (43 L/m^2^·h and 32 L/m^2^·h, respectively) significantly greater than the fluxes obtained with the XLE membrane (30 L/m^2^·h and 16 L/m^2^·h initial and final flux, respectively). Contrary to the dead-end mode results, the flux decline with the NF90 membrane was rather modest (Figure S4), despite the increase of the feed solution osmotic pressure. The highest concentration of PO_4_-P was achieved with the NF90 membrane (concentration factor, C_f_ = 4.2) in comparison to that of XLE (C_f_ = 3.1). With the NF90 membrane, the NH_4_-N concentration in the retentate was also somewhat greater (C_f_ = 2.2, compared to C_f_ = 2.0 for XLE) (Figure S5a,b). The rejection of nutrients (Figure S5c,d) remained high during all tests, with the highest values measured for PO_4_-P (98.5% and 97.1% with XLE and NF90, respectively), while NH_4_-N rejection was similar for both membranes (~83%). Additional comparative tests with the two membranes were performed, using as feed real FLD, as follows.

##### Performance Tests with Untreated FLD

The detailed composition of FLD samples (directly obtained from the operating plant) for these tests, listed in Table A1, Appendix A, exhibits significant temporal variability. In general, it should be noted that, although this FLD is characterized by rather low organic load (TOC: 19.7–69.6 mg/L), moderate inorganic content (TDS: 2670–3750 mg/L), and manageable amount of nutrients (NH_4_-N: 168.4–211.7 mg/L and PO_4_-P: 74–134 mg/L), compared to other similar FLD in the literature [38,56,57,58], it neither meets the regional quality standards for disposal (to a nearby river [59]) nor the national requirements for reuse [41]. Moreover, although the FLD nutrient content is close to the corresponding concentration of the feed solutions of the previous section (~150 mg/L for NH_4_-N and PO_4_-P, Table A2), the values of electric conductivity (eC = 4170–5860 μS/cm) and alkalinity (1515–2439 mg CaCO_3_/L) are significantly higher than those of the synthetic solutions (~2000 μS/cm and zero alkalinity, respectively). 

The experimental conditions in these tests were similar to those employed with the synthetic solutions (sub-Section ‘Permeability and rejection tests with synthetic solutions’); i.e., constant pressure 5 bar, cross-flow velocity 20 cm/s, two parallel test sections, and recirculation of feed solution/retentate. Between the FLD samples received (Table A2, Appendix A) and those analyzed as feed solutions in the experimental tests (Table A3), a slight dilution took place, attributed to an amount of water remaining in the experimental set-up due to prior cleaning. Performance tests with max. ~40% permeate recovery were performed, using XLE and NF90 membranes for the treatment of sample S1 (Table A1). The results confirmed that both membranes exhibit similar performance regarding nutrient rejection, with NF90 performing somewhat better in terms of flux, as one would have expected. In particular, NF90 exhibited higher initial flux (27 L/m^2^·h) than XLE (20 L/m^2^·h), resulting in reduced processing/test time (600 min), compared to that needed by XLE (702 min) for nearly the same total permeate recovery, as shown in Figure S6. 

It should be noted, however, that the level of fluxes and of rejection measured in these tests with FLD feed is substantially smaller, compared to that obtained with equivalent synthetic feed solution. This is attributed to fluid-quality differences between the two feeds; in particular, to the smaller TDS (i.e., osmotic pressure) as well as the absence of foulants in the synthetic feed solution. Furthermore, both membranes, especially NF90, exhibited a significant flux decline, especially near the end of experiments (as shown in Figure S6), which is attributed to the concentration of the feed solution and also to membrane fouling/scaling phenomena, mainly of inorganic nature. Flux decline, due to fouling of NF90 and BW30 membranes, was also observed by Magalhães et al. [26] mainly attributed to surface roughness of membranes. The quite significant level of total alkalinity (expressed as mg CaCO_3_ per liter) in all FLD samples (Table A1), in combination with the present divalent cations of calcium (Ca) and magnesium (Mg), favor precipitation of CaCO_3_ and phosphate salts. Indeed, speciation calculations of the feed and retentate solution (at permeate recovery ~40%), obtained with PHREEQC software (test 1P, Table A3), indicate that Ca phosphates as well as Ca and Mg carbonates have a tendency to precipitate since their concentration exceeds the saturation limit (SI > 0).

**Figure 5:**
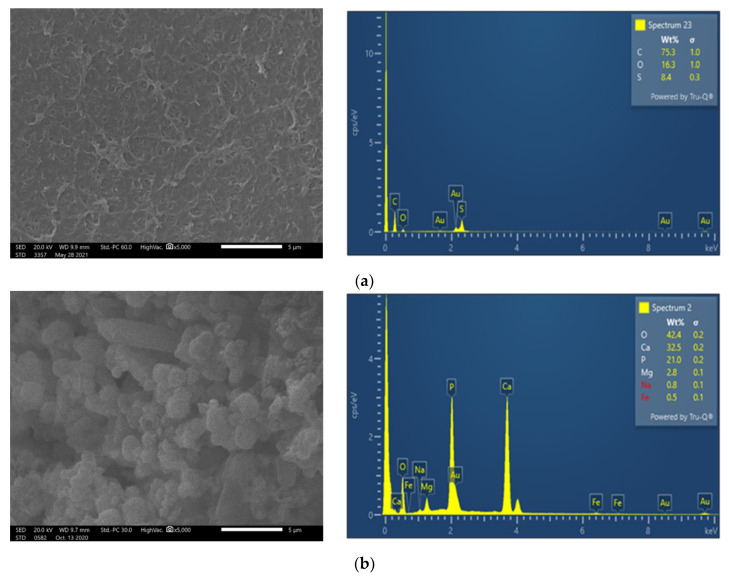

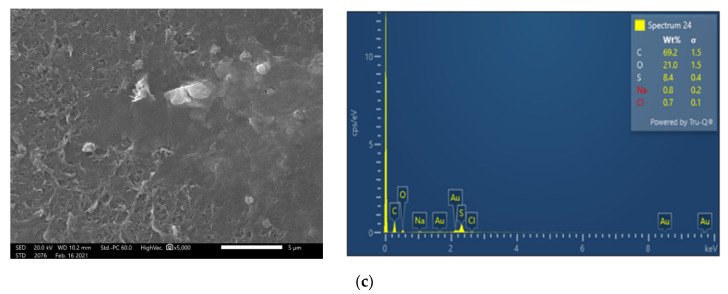
SEM images (×5000) and EDS spectrum of membrane surface. Comparison of (**a**) fresh NF90 membrane; (**b**) NF90 after the filtration of untreated FLD (test No 1P); and (**c**) after the filtration of pretreated FLD (test No 3P).

Membrane surface characterization was also performed, employing SEM and EDS analysis, to examine membrane condition after filtration of untreated FLD (Figure 5b). The SEM image exhibits significant deposits with a rather high content of Ca and P, at 32.5 wt% and 21.0 wt%, respectively. Moreover, although the coexistence of carbonate salts could not be confirmed using the available EDS analysis, it is known that the feed solution alkalinity favors sparingly soluble salt precipitation and membrane scaling. Finally, in line with this explanation is the observed reduced P (as well as Ca and Mg) concentration in the retentate of test 1P (C_f_: 0.9 < 1.0), suggesting loss of PO_4_^3−^ ions, likely through precipitation.

Hence, the NF90 membrane was selected, over XLE, for further investigation, using real FLD samples, in the following process development stage. Moreover, the investigation of feed solution pretreatment was considered necessary to address the alkalinity issue. The need for pretreatment was also stressed by Sheets et al. [13]. 

##### Performance Tests with Pretreated FLD

Two pretreatment approaches were followed:

(a) In tests 3P and 4P (Table A3), the feed pH was controlled at 6.5 throughout the filtration process with periodic addition of sulfuric acid (6N) that reached approximately 0.05% of the total feed volume.
(b) In test 2P, part of the bicarbonate and ortho-phosphoric ions of FLD were precipitated with Ca(OH)_2_ addition. The supernatant was also acidified with H_2_SO_4_ due to its high pH; this is an alternative pretreatment process path for possible hydroxyapatite recovery [29] and membrane scaling mitigation.

Pretreatment through pH adjustment of the feed solution (Tests 3P–4Ρ, 5 bar with fresh NF90 membrane) resulted in increased retention of NH_4_-N (C_f_ 2.2–3.2) and PO_4_-P (C_f_ 2.4–2.7) for permeate recovery of 55.5–69.5% (Table A3, Appendix A). This result clearly suggests that reducing/controlling the feed solution alkalinity is critical for efficient nutrient retention and increase in permeate recovery. The alternative pretreatment step, for phosphate precipitation with Ca(OH)_2_ prior to test 2P, appears to be rather unnecessary, since no improvement was observed, neither in terms of flux, nor in nutrient rejection/retention compared to tests 3P–4P, where only feed pH adjustment took place. On the contrary, the possible precipitation of phosphate salts due to the Ca(OH)_2_ addition resulted in the reduction of phosphate content of both feed solution and retentate.

Among tests 2P–4P, test 4P exhibited the best performance with higher flux and permeate recovery up to 70% (Figure S7), possibly attributed to the lower inorganic content of the feed solution (3880 μS/cm), compared to that of tests 2P and 3P (~4900 μS/cm). Nevertheless, flux values were almost 50% lower than those observed in cross-flow tests with dilute synthetic solutions at similar test conditions. This fact is attributed to the increased osmotic pressure of FLD, which led to lower effective transmembrane pressure ΔΡ (i.e., ΔΡ = P_applied_ − P_osmotic_) of the process. As also shown in Figure S7, the flux decline in tests 1P–4P is typical for batch processes where concentration polarization and/or fouling phenomena take place [60,61]. 

SEM images and EDS analysis show that on the NF90 membrane surface, after the pretreated FLD filtration (test 3P), there are rather limited/localized deposits (Figure 5c), in comparison to the fresh membrane surface (Figure 5a). In contrast, the membrane specimen from test 1P (Figure 5b), after untreated FLD filtration, is loaded with deposited/scale particles, mainly comprised of Calcium Phosphate salts. This observation is supported by the respective EDS data (where Ca, P are dominant) as well as by the related supersaturation indices (listed in Table A3), which were determined on the basis of retentate composition. The above SEM/EDS data clearly suggest that the FLD-feed pretreatment/pH adjustment is effective in mitigating membrane fouling. 

In Table S5, results of the membrane’s foulant/deposit *dissolution tests* are presented, which also support the effectiveness of the pretreatment/acidification. Specifically, the determined mass of Ca, Mg, P compounds on the membrane surface is significant after filtration of untreated FLD (test 1P) but insignificant after pretreated FLD (tests 2P, 3P). This mass of scalants most likely comprises Ca and Mg phosphate and carbonate salts. Indeed, by inspecting the speciation of the treated FLD solutions of tests 2P-4P (Table A3), undersaturation of these salts is observed, except for hydroxyl-apatite (HA). The retentate of test 2P (at recovery ~40%) is undersaturated in most of these salts (except HA), due to the rather low level of alkalinity and orthophosphates, removed by precipitation. The retentate of test 3P (at recovery ~40%) is weakly saturated with phosphates, including Ca_3_(PO_4_)_2_(beta), Ca_4_H(PO_4_)_3_:3H_2_O, and HA, but they seem to have rather insignificant effect on membrane scaling (Figure 5c) and flux decline (Figure S7). This trend may be also favored by the fair hydrodynamic conditions in the spacer-filled membrane channels and the permeate flux. Regarding TOC, the measured mass surface density is low and roughly the same for tests with untreated and pretreated feed (63–87 mg/m^2^), indicating that organic fouling is relatively insignificant.

The preceding membrane performance results (including fluxes and fouling propen-sity) clearly demonstrate the effectiveness of the FLD-feed pretreatment/pH-adjustment, which is accepted as a necessary processing step in further overall process development. 

#### 4.2.2. Comprehensive Investigation of Membrane Process Performance

##### Selection of Experimental Conditions through Process Simulation

For further experimental investigation of membrane process performance with pretreated FLD feed, more realistic test conditions were determined, by employing appropriate software, simulating SWM module performance. Within such a relatively narrow range of selected test conditions, the optimal process-parameter values are expected to be located, in order to design an appropriate pilot unit, as further discussed.

Two simulators were used; i.e., the commercial software of DuPont™ (Water Application Value Engine—*WAVE*, v. 1.82.824) [62] and another (designated as *SWM/NRRE*) developed in the authors’ laboratory for simulating SWM element performance [63,64]. Preliminary simulations were performed for a fair-size pilot system, of one pressure vessel with four SWM elements (each of 2.6 m^2^ active surface, membrane type ‘NF90 2540’) and permeate recovery of ~50%. The typical operating conditions of test No 4P were selected as input data for the simulations (Table S6, Supplement). 

The simulation results, regarding main process parameters, are included in Table 1. According to the WAVE results, the feed pressure in such an NF system should be 9.0 bar to obtain an average 54.1 L/m^2^·h of permeate with 125.2 mg/L of TDS. Additionally, the WAVE output for the simulated system included permissible process-parameter limitations/warnings, as listed in Table S7; i.e., for permeate flow (≤1 L/min) and recovery per element (≤12%). However, it should be stressed that using the WAVE software, the nominal NF90 membrane permeability is a fixed (unknown) value and cannot be modified by the user. Moreover, if the effective permeability (K_p_) is calculated using the WAVE output (i.e., the permeate flow of the entire element, the inlet pressure, and osmotic pressure), the resulting K_p_ value is 8.3 L/m^2^·h·bar, whereas a more realistic initial K_p_, determined experimentally here (Section 4.2.1, test 4P), is significantly lower (i.e., K_p_ = 7.1 L/m^2^·h·bar for fresh/pristine membrane).

The NRRE/SWM simulator predicts a required feed pressure of 9.4 bar, applied at the pressure vessel entry, with a permeate TDS 69.1 mg/L and average flux 39.0 L/m^2^·h for the first element. These flux values (Table 1) are closer (compared to WAVE output) to those obtained during the cross-flow tests with NF90 membrane using synthetic solutions at 5 bar (Figure S4), whereas the estimated quality of permeate per element is within the acceptable limits for disposal (<450 mg/L TDS) [59]. Consequently, these simulations suggest that a realistic constant feed pressure of a membrane process with ~50% recovery (further investigated here) would be at about 8 to 10 bar. 

##### Comprehensive Membrane Performance Tests

The following tests (cross-flow FLD filtration at 8–10 bar) also involved the implementation of three different cleaning protocols to assess the degree of reversibility of potential membrane fouling. These tests aimed at determining near optimum operating conditions, membrane performance parameters, and cleaning protocols for further process development.

The tests (Table A4) were grouped in experimental series designated as #1 to #4 and involved three consecutive rejection tests with 40-50% recovery. After each rejection test, membrane cleaning was performed as follows: (i) 1st test, flushing with DI water (DI); (ii) 2nd test, chemical cleaning (clean in place—CIP), suggested by the manufacturer [65]; (iii) 3rd test, flushing with DI water (DI). Membrane conditioning and clean water permeability measurement took place prior to each rejection test (detailed protocol in Section S5 of Supplement). In the test series #3_9 bar, the CIP was replaced by flushing the system with the produced permeate (PERM).

As shown in the data listed in Table A4, the clean water permeability of the fresh, unused membrane NF90 varied between 9.0 and 10.9 L/(m^2^·h·bar) in accord with the value (10.3 L/m^2^·h·bar) provided by the manufacturer [46]. Evidently, after the first rejection tests (#1-#4a_FM) with the acidified FLD and the subsequent flushing of the system with DI, the clean water permeability was reduced by ~35%, i.e., in the range of 6.3–7.1 L/m^2^·h·bar, suggesting degradation of the fresh membrane initial properties, likely due to irreversible fouling. The second rejection tests and the ensuing CIP resulted in either similar (test #2c_CIP) or slightly higher (tests #1c_CIP and #4c_CIP) clean water permeability values.

**Figure 6:**
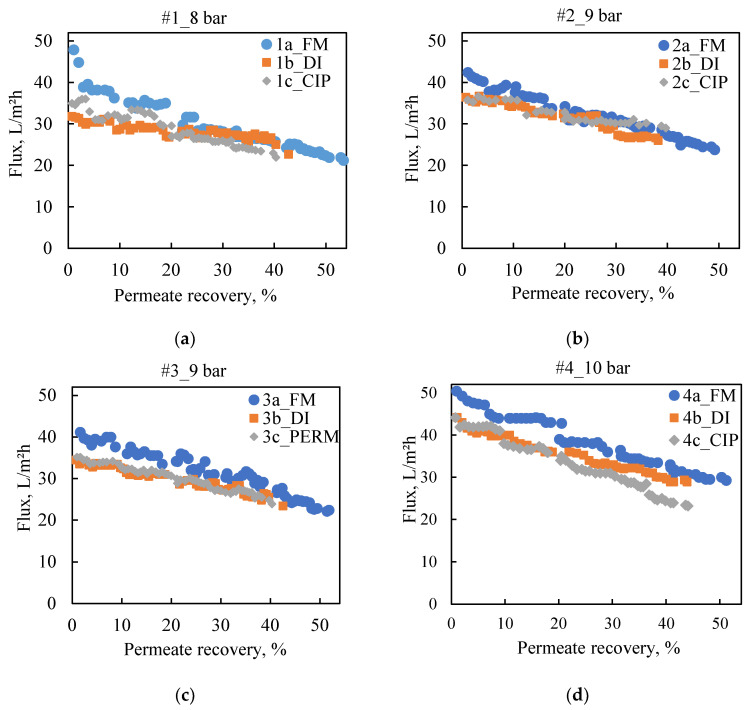
Flux variation versus permeate recovery of the test series #1-#4. Panels: (**a**) Feed S3 (#1_8 bar); (**b**,**c**) Feed S5 (#2_9 bar & #3_9 bar); and (**d**) Feed S4 (#4_10 bar).

Regarding the flux variation (Figure 6) of the rejection tests, the increase of the ap-plied pressure from 8 to 10 bar resulted in increased permeate flow as expected from the simulations. The highest initial flux was observed in test #4a_FM with applied pressure of 10 bar. As with clean water permeability, the initial fluxes of the second and third rejection tests were reduced, although the respective flux profiles were relatively smooth in all cases. Hence, the tests with the fresh membranes (#1a_FM to #4a_FM) are representative of the first operating period of a real NF system, when conditioning of the membrane takes place, whereas the subsequent tests (i.e., #1-#4b_DI and #1-#4c_CIP/PERM) may correspond to the smoother longer-time operation of the system. No significant difference was observed regarding the initial and final flux of the tests, which followed CIP and flushing with NF permeate, suggesting that the flushing with NF-permeate could be almost equally effective in cleaning the NF90 filtration system (perhaps on a daily/regular basis), as the chemical agents of CIP.

It should be also noted that the flux reduction (for pretreated FLD and various feed pressures), depicted in Figure 6, or in terms of normalized flux in Figure S8, is practically linear, particularly if plotted versus permeate percent recovery. As indicated by the comparison and agreement of these data with the process simulation results (in following Section 4.3.1), such linear variation can be mainly attributed to the increasing osmotic pressure of the retentate with increasing recovery, thus leading to reduced transmembrane pressure.

The difference between the *clean water permeability* and the *effective* membrane perme-ability during operation, could serve as an indication of the degradation of membrane productivity due to fouling [61]. As shown in Table A4 (Appendix A), the effective permeability K_p_ = J/(ΔP – Δπ) of the compacted fresh NF90 membrane (tests #1-#4a_FM), was 6.1–6.7 L/m^2^·h·bar whereas during the following rejection tests, it was modestly reduced to 5.4–5.7 L/m^2^·h·bar. Therefore, the fouling caused on the NF90 by the acidified FLD at laboratory scale could be characterized as rather mild and reversible, since:

- The flushing of the system with DI and/or with NF permeate resulted in slightly reduced membrane permeability, similar to that achieved after CIP.
- The filtration tests were repeatable in terms of the flux profiles.

**Figure 7:**
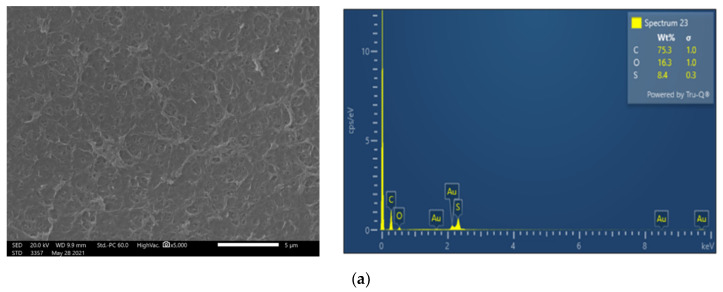

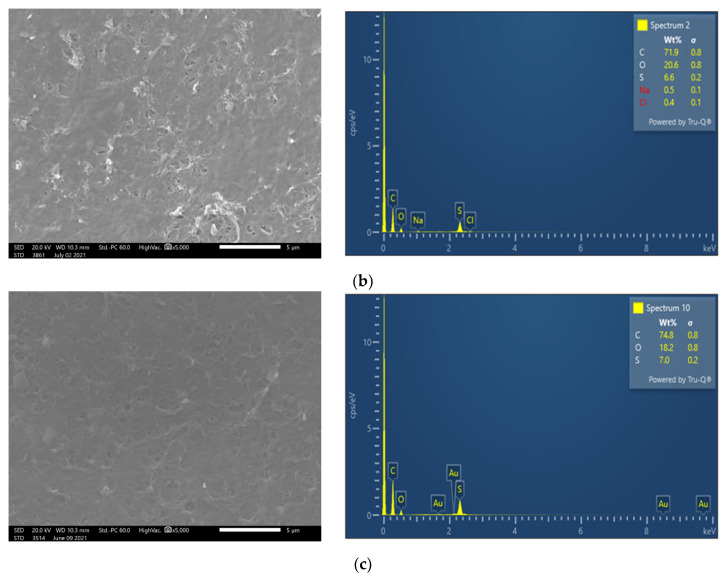
SEM images (×5000) and EDS analyses of membrane surface (**a**) fresh NF90; (**b**) NF90 from the 1st test section right after the test 3c_PERM; and (**c**) NF90 from the 2nd test section after the test 3c_PERM and flushing of the experimental set-up with DI water.

To investigate fouling and cleaning effectiveness in more detail, the membranes of the two test sections were treated differently at the end of the third rejection test (#X_CIP/PERM). The specimen from the 1st in the row test section was obtained right after the test, whereas the one of the 2nd test section was removed after flushing of experimental set-up with DI water. Both membranes were analyzed via dissolution tests (Section 3.1). The estimated surface density of fouling/scaling species for each membrane coupon is included in Table 2, where it is evident that the TOC content is significantly reduced after DI water flushing. The same effect is also observed for the inorganic species of phosphorus, calcium, magnesium, and sodium between the two test sections of each series. These results in comparison with the SEM images of the unused NF90 membrane (Figure 7a) and of the respective specimens obtained right after test #3c_PERM (Figure 7b) or after the subsequent system flushing with DI water (Figure 7c) confirm the rather insignificant fouling propensity of the acidified FLD. Moreover, flushing of the NF system with DI water appeared to be efficient in removing a significant part of membrane deposits.

The species rejection performance of the NF90 membrane was also not altered among the various tests of each experimental series and remained at relatively high levels, as clearly shown in Figures S9 and S10. Considering the detailed characterization of the composite permeate and retentate produced in each test (Table A4), the following general assessment is made regarding the potential utilization of the two main streams of the process:

- The *NF retentate* (ΝH_4_-Ν: ~250–450 mg/L and PO_4_-P: 110–160 mg/L at permeate recovery ~50%) is considered useful as a possible liquid fertilizer, compared to respective products of the literature with similar physicochemical properties [26,38], although dilution may be needed [21] to render it appropriate for soil fertilization [66]. Alternatively, the recovery of the retentate nutrients could be achieved, e.g., through the precipitation of phosphate salts such as struvite (Magnesium Ammonium Phosphate, MAP, MgNH_4_PO_4_·6H_2_O), which is a well-known slow-release fertilizer [24,67].
- *NF permeate*: The NF90 membrane exhibited high nutrient rejection (approx. 97% for NH_4_-N and 99% for PO_4_-P) in all tests, as shown in Figure S10, resulting in rather low concentrations in the collected composite permeate (NH_4_-N: 3–7 mg/L and PO_4_-P: 0.2–1.5 mg/L at permeate recovery 40–50%). Furthermore, pH and eC varied between 6.1–7.2 and 81.5–199.6 μS/cm, respectively. Generally, the NF permeate can be characterized as clean, transparent, and satisfying the regulations for water reuse in restricted irrigation [41]. However, the currently in-effect, rather stringent local regulations [59] regarding ΝH_4_-Ν (≤2 mg/L) do not allow permeate disposal.

In summary, comprehensive results were obtained from laboratory tests, regarding the performance of NF membrane process for FLD treatment, including determination of effective membrane permeability as well as assessment and related data on membrane fouling/scaling and on mitigation measures. This information permits us to proceed further with process development, as follows.

### 4.3. Towards Process Scale-Up—Pilot Plant Design

#### 4.3.1. Projections of Real/Pilot Plant Performance—Process Simulations

The proposed methodology was validated by comparing selected experimental data from Section 4.2.2 with respective simulation results of performance of SWM modules. Furthermore, basic design data were obtained for an appropriate industrial membrane pilot unit. The NRRE/SWM software [63,64,68] was selected (over WAVE) due to its capability of using/imputing realistic membrane effective permeability values (for the particular feed fluids) experimentally determined. Specifically, the input data for these simulations (Table S8) were obtained from the results of the experimental series #3_9 bar (tests 3b_DI and 3c_PERM). Key features of the simulated system are as follows:

- 2 to 4 SWM modules (NF90- DuPont) in a pressure vessel, depending on the desired permeate % recovery.
- Active membrane surface area per SWM module 2.6 m^2^ (Commercial type NF90 ‘2540’—DuPont).
- Simulations for system/pilot operation under constant recovery of: (i) 20% (two membrane elements in series, Case No 1) and (ii) 40–44% (four membrane elements in series, Cases No 2–No 4).

**Figure 8:**
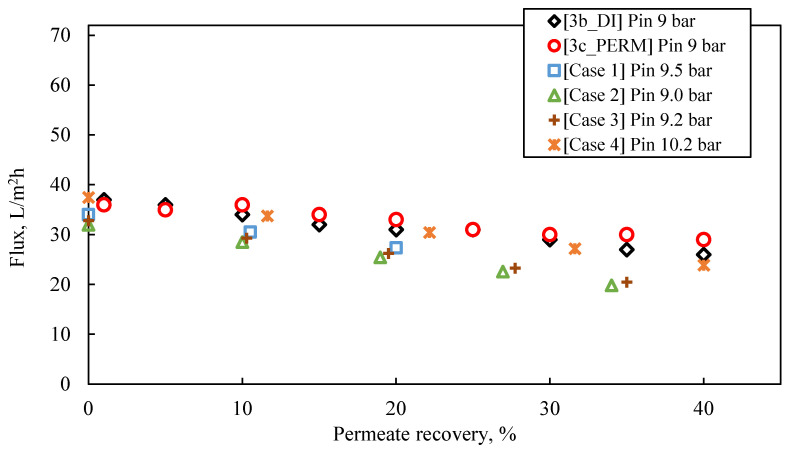
Experimental data (tests #3b_DI and #3c_PERM) of permeate flux temporal variation, compared with results obtained from SWM simulator (Cases 1–4); Feed: S5.

**Figure 9:**
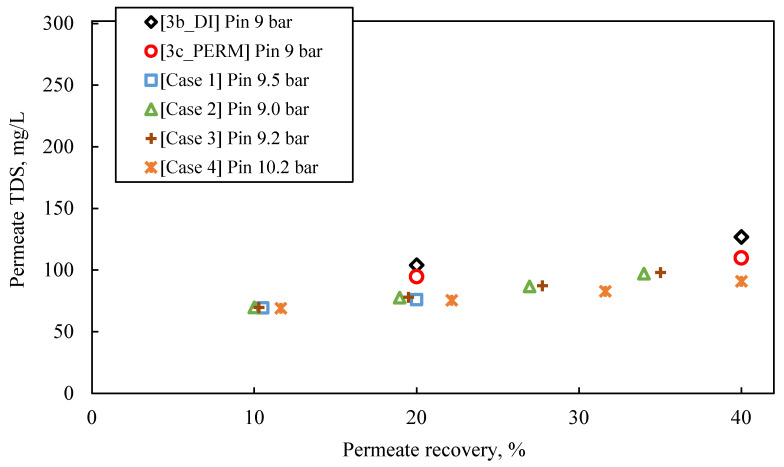
Experimental data (tests #3b_DI and #3c_PERM) on variation of permeate quality in terms of TDS, compared with results obtained from SWM simulator (Cases 1–4); Feed: S5.

**Figure 10:**
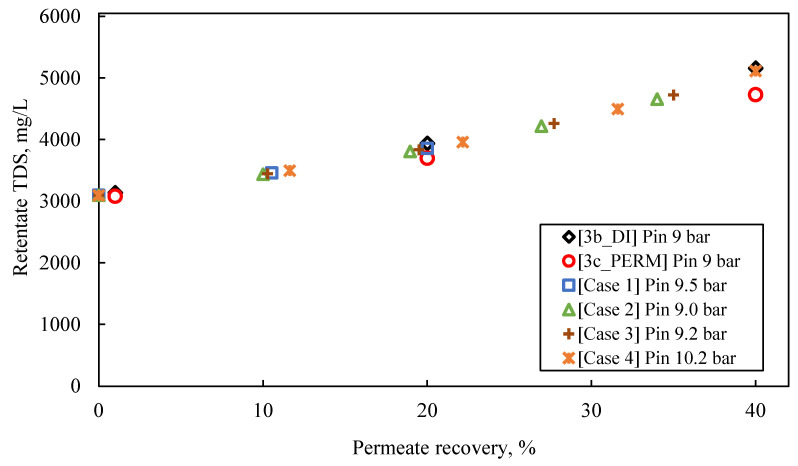
Experimental data (tests #3b_DI and #3c_PERM) on variation of retentate quality in terms of TDS, compared with results obtained from SWM simulator (Cases No 1–4); Feed: S5.

The following observations are made by inspecting the data of the simulated pilot system performance (i.e., permeate flux and TDS, retentate TDS), summarized in Table S9 and in Figure 8, Figure 9 and Figure 10, as well as their comparison with the results of experimental series #3:

- For an input inlet cross-flow velocity of 0.22 m/s, the feed flow rate is 13.3 L/min (i.e., ca. 798 L/h or 19,152 m^3^/d).
- For permeate recovery of 40% from the FLD membrane treatment (Figure 8), the prediction by the simulator inlet pressure (of ~9.0–10.0 bar), compared to the applied pressure in the laboratory cross-flow tests (i.e., 9.0 bar), is generally considered satisfactory, at this stage of process development.
- In the case of a pilot with two membrane modules (Case No 1) and permeate recovery of 20%, the simulation result regarding initial flux is 32 L/m^2^·h, with inlet pressure of 9.5 bar.
- Simulation Case No 4 (four SWM elements in series and permeate recovery 44.4%) generally provides the best fitting to the experimental data (as shown in Figure 8). The experimental values of initial flux (37 L/m^2^·h and 36 L/m^2^·h for the tests #3b_DI and #3c_PERM, respectively) are very close to those of the simulation. However, the predicted feed pressure from the simulation (10.2 bar) is somewhat greater (by ~10%) compared to the applied one in the laboratory cross-flow test (9 bar).
- The simulated flux variation (in case No 4 and others, where no fouling is considered) is very similar to that of experimental results, indicating mild/controllable fouling of the membrane surface.
- The predicted quality of the composite permeate in terms of TDS (Figure 9) is comparable with that of test results, clearly satisfying the respective regulations for water reuse (i.e., <450 mg/L) [2,41]. Excellent agreement is observed between simulations and experimental data regarding retentate concentration variation with % recovery (Figure 10).

In summary, the comparison between simulations and experimental data, obtained from realistic cross-flow laboratory tests, is considered satisfactory, thus allowing us to proceed further and employ the collected information for the design of an industrial size pilot plant, toward developing the process to higher TRL.

#### 4.3.2. Pilot Plant Basic Design

Based on the validated process model/simulation results, the following basic design data are obtained for an appropriate industrial pilot unit, to treat FLD:

- Membrane module type: NF90 2540 (DuPont).
- Main feed pump max. pressure: ~15 bar.
- Capacity of pilot unit: Feed flow rate ~15 L/min or ~22 m^3^/day for 24 h/day operation.
- Typical superficial cross-flow velocity at inlet of 1st pressure vessel: 20–25 cm/s.
- Targeted permeate recovery: 20% to ~60%, depending on operating mode.
- Feed pretreatment, i.e., controlled acidification of FLD and prefiltration with cartridge filter.
- Provision for membrane periodic cleaning: (i) Flushing with permeate and (ii) CIP facility.

The above basic design data have served for the detailed engineering design and construction of the industrial pilot plant, briefly described as follows.

### 4.4. Pilot Plant Preliminary Tests

#### 4.4.1. Pilot Brief Description

A view of the fully instrumented pilot, with automatic control and data acquisition, including provisions for sampling of the process streams, is presented in Figure S11a in the Supplemental Materials. The pilot plant comprises two pressure vessels in series (marked as *Stage 1* and *Stage 2*). Two NF90-2540 (DuPont) SWM modules (of active area 2.6 m^2^ per module) are placed in each pressure vessel; thus, the total active membrane area of both vessels is 10.4 m^2^. The feed is pumped from the feed tank (1000 L) to the pressure vessels with a horizontal multistage pump (1HM22N11T, LOWARA, Xylem Inc., USA), whereas the permeate and concentrate streams can be collected in two 1000L vessels. The pilot is also equipped with an auxiliary vessel for pH adjustment (through PID control) and a 5 μm cartridge filter for feed pretreatment. Additionally, a clean-in-place (CIP) system is installed with respective vessels marked as *Acid* and *Alkali*, as shown in Figure S11b. The membranes can be also flashed/cleaned with permeate. Process parameters (pH, eC, temperature, flow rate, and pressure) of various streams can be controlled/monitored and recorded. Supervision and control of the pilot plant is performed through a human–machine interface (HMI) and a touch screen (MT8090XE, WEINTEK, Taiwan) (Figure S11b).

The plant can be operated in various modes, including continuous once-through, batch with total concentrate- and permeate-stream recycling (simulating continuous operation), or a batch with only concentrate-stream recycling. For the feed fluids treated, the system is designed to attain permeate flux at the level of ~30 L/m^2^·h. Thus, employing four NF90-2540 SWM modules in series, under typical operating conditions, for feed flow rate ~15 L/min, the concentrate and permeate flow rates are ~10 L/min and ~5 L/min, respectively, leading to a permeate recovery of 30–35% per pass.

#### 4.4.2. Preliminary Pilot Tests

For preliminary assessment of pilot/process performance, tests were conducted with a rather simple synthetic solution (to economize chemicals) comprising single- and bi-valent ions, as shown in Table S10. These tests were conducted with total permeate and concentrate recycling to the feed vessel, for simulation of continuous (once through) operation; therefore, the concentration in the feed vessel remained practically constant. Tests were carried out with either one pressure vessel (*Stage 1*, 2 SWM modules), or two vessels in series (total 4 SWM modules). Cleaned membrane was used in all tests. The inlet pressure and feed flow rate were maintained constant at ~5.7 bar and 13 L/min, respectively; the latter corresponds to inlet cross-flow velocity (at 1st SWM entry) u_in_ = 0.21 m/s). Table S11 includes the main experimental conditions and input parameter values for the simulations with NRRE/SMW software [34,35].

**Figure 11:**
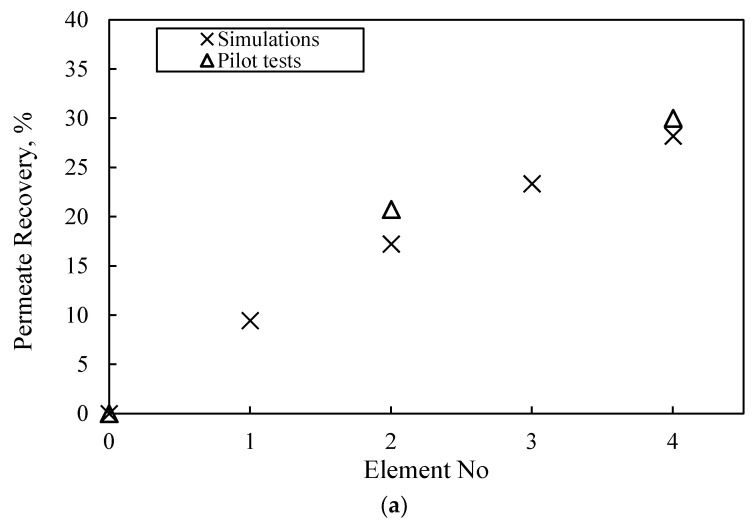

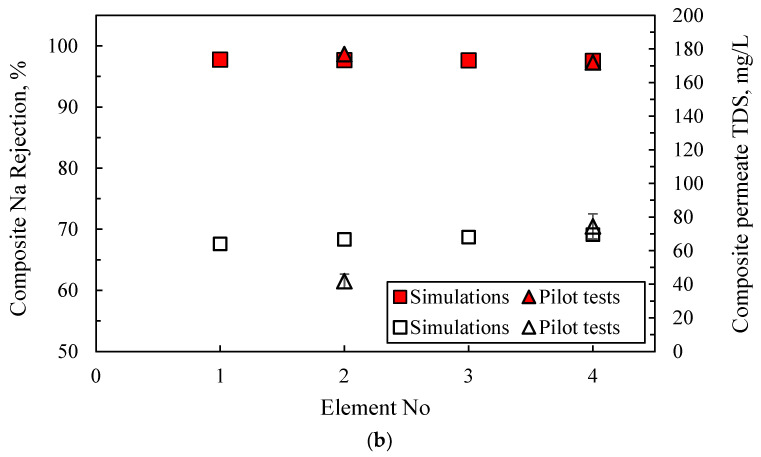
Axial variation (along 4 SWM modules in series) of NF membrane process parameters. Comparison of pilot test data with simulation results at the exit of each SWM module. (**a**) Permeate % recovery and (**b**) permeate TDS concentration and Na rejection.

Table S10 includes the measured composite permeate and concentrate characteristics, at the exit of the first and second stage (i.e., at the exit of second and fourth SWM modules, respectively), which are satisfactory and in line with the results of the laboratory-scale tests. It is noteworthy that the achieved rejection of single- and bi-valent ions (>97% and >99%) and permeate recovery (~30%) for once-through operation are high, as expected. Additionally, Figure 11 presents the variation of key NF process parameters along the four SWM modules (in series) of the pilot unit. The depicted comparison of the pilot test data (i.e., permeate % recovery and permeate TDS concentration) with simulation results at the exit of each SWM module is also considered satisfactory, thus confirming the reliability of the model/simulator. Therefore, the successful trial operation of the pilot plant and the validation of the NRRE/SMW simulator warrant their further use for process scale-up and optimization.

## 5. Comments

Aiming to address the main process targets, a systematic methodology was implemented, which relies on the following sequence of experimental steps, in conjunction with advanced membrane process simulations.

*Dead-end tests.* These preliminary tests, performed with synthetic solutions of typical FLD composition, aim to screen candidate membrane types and select appropriate ones for further detailed testing. Collected key parameter data (flux, permeate/retentate concentration, and species rejection) are presented/interpreted as a function of the % permeate recovery.

*Cross-flow tests, combined with process simulations.* These tests, in an appropriate laboratory set-up, are fairly representative of conditions encountered in practice, as test sections with spacer-filled channels and a high-pressure pump are employed. A fairly large initial feed fluid volume (6–8 L) is used (considering the limited filtering membrane surface area), to permit long-time tests, as needed. “Once through” feed fluid tests can be performed in this set-up. However, the data for this study were collected mainly in a “batch” operating mode, whereby permeate is continuously removed, and the concentrate is recirculated; consequently, the feed concentration is steadily increasing. Again, the key process-parameter data are presented as a function of % permeate recovery. It should be stressed that such data are quite representative of specific *local conditions* encountered along a membrane pressure vessel, where the feed/retentate is continuously concentrated in the axial flow direction. Therefore, these experimental process-parameter data, *plotted versus % permeate recovery*, are compared to respective simulation results of a real membrane process, i.e., for flow along multiple SWM modules included in a pressure vessel. Validation of the advanced model/simulator, in this manner, allows realistic projections for process scale-up purposes.

Through this systematic approach, an appropriate pilot plant was designed and built (for operation in the industrial environment) and a relatively narrow range of near-optimum operating conditions was specified for testing. Satisfactory pilot performance was obtained in preliminary tests with synthetic solutions. The proposed approach is contrary to what is usually encountered in relevant literature (e.g., [18,38]), where ad hoc design of pilot units is reported for such investigations.

## 6. Conclusions

Valorization of anaerobic digestate liquid effluents is investigated herein, by implementing a systematic methodology for the development of a membrane process scheme. A typical liquid digestate from an industrial plant is employed. The methodology involves a sequence of specially designed laboratory experiments, in conjunction with reliable membrane process simulations. Through such experiments, the membrane process performance is investigated in detail, including determination of key membrane process parameters (i.e., effective membrane permeability for the specific feed fluid) as well as quantitative assessment of other important issues (i.e., membrane fouling/scaling and cleaning). The main experimental data are presented in terms of the % permeate recovery, which is monitored during the membrane separation tests. This is a notable feature of this study, regarding the presentation and interpretation of experimental data, which allows their direct comparison with similar membrane process simulation results and facilitates method validation and development. As such, this methodology is of general applicability to a class of similar membrane-based process development tasks.

The process presented here for FLD involves NF membranes yielding permeate/water (i.e., the largest portion of the treated liquid digestate) of quality appropriate for reuse in industry and/or in restricted irrigation as well as a concentrate (containing the P, N nutrients) for possible utilization in fertilization. Based on the laboratory-scale results, a pilot plant was designed and built, and it underwent preliminary testing successfully. In the following stage of process development, data from this pilot plant operating in the industrial environment will be employed to optimize the membrane separation and further examine the valorization of concentrate (obtained from this pilot) through precipitation/separation of the nutrients in the form of struvite or in liquid fertilizers. Such work at pilot scale is already in progress.

## Figures and Tables

**Table 1:** Computational input and output data of WAVE and SWM/NRRE simulators for permeate recovery 50%; Feed: S3 (Table A3). Four SWM elements in series.

SWM Element	Feed Pressure, bar	Feed Flow, L/min	Permeate Flow, L/min	Average Flux, L/m^2^·h	Recovery, %	Permeate TDS, mg/L	Feed TDS, mg/L
WAVE Simulator							
1	9.0	15.5	2.3	54.1	15.1	125.2	3069
2	8.6	13.2	2.0	47.8	28.0	163.9	3586
3	8.3	11.0	1.8	41.5	39.8	218.3	4217
4	8.1	9.3	1.5	35.3	49.5	296.3	4982
SWM/NRRE Simulator							
1	9.4	12.1	1.8	39.0	15.1	69.1	2930
2	9.1	10.3	1.6	34.3	28.5	81.5	3440
3	8.9	8.6	1.4	29.3	40.2	97.1	4070
4	8.7	7.2	1.2	24.0	50.0	116.6	4846

**Table 2:** Estimation of foulant species surface density; dissolution tests of NF90 membrane specimens after the test series #1–#4 with HCl 60 mM and NaOH 25 mM.

Test Series	#1_8 bar		#2_9 bar		#3_9 bar		#4_10 bar	
Test section	1st *	2nd **	1st *	2nd **	1st *	2nd **	1st *	2nd **
Species	Estimated species surface density on NF90 membrane, mg/m2							
TOC	199.3	81.2	169.0	77.4	131.6	65.2	287.9	81.0
P	22.4	ND	145.8	40.9	13.2	ND	25.4	ND
Ca	51.9	50.2	278.7	83.8	300.4	2.7	20.3	7.1
Mg	8.0	1.4	11.0	3.4	56.0	8.4	10.2	1.1
K	44.7	14.7	ND	ND	33.1	14.5	47.0	3.9
Na	264.7	169.5	195.1	170.3	259.4	179.8	170.7	74.7

ND: Not detected/insignificant; * NF90 membrane from the first test section right after the test; ** NF90 membrane from the second test section after the test and flushing of the experimental set-up with deionized water.

## Data Availability

The data presented in this study are available on request from the corresponding authors.

## Supplementary Materials

The following supporting information can be downloaded at: https://www.mdpi.com/article/10.3390/membranes13030297/s1. Table S1. Specifications and performance data of the commercial membranes (2540 modules) used in preliminary dead-end filtration tests; footnotes indicate manufacturers’ tests conditions. Table S2. Instruments used for characterization of membrane performance in the cross-flow filtration tests. Table S3. Comparison of nutrient rejection and permeate flux using single-nutrient and “binary” synthetic feed solutions, in dead-end filtration mode; applied pressure 5 bar. Table S4. Results of the rejection tests performed in the cross-flow set-up with XLE and NF90 membranes. Feed: Synthetic solutions, applied pressure ~5 bar, ~25 °C, cross-flow velocity ~20 cm/s. Table S5. Foulant species mass surface density on NF90 membrane (preliminary tests 1R-3R at 5 bar). Dissolution technique employed, involving HCl 60 mM and NaOH 25 mM; average values from both test sections (1 and 2). Table S6. Input data for the Simulators WAVE (DuPont) and NRRE/SWM, corresponding to S3 feed solution characteristics. Table S7. WAVE Simulator operation limits (design warnings) for the input data of Table S6. Table S8. Input data for a small/middle scale pilot plant simulation with NRRE/SWM simulator (test series #3_9 bar with pretreated FLD). Table S9. Simulations of a small/middle scale pilot plant with NRRE/SWM simulator (input data from Table S8). Table S10. Composition of synthetic feed solutions, NF permeate, concentrate and rejection results of pilot tests. Table S11. Experimental conditions and input data for pilot test with synthetic solution under constant pressure. Figure S1. Experimental set-up, involving pressurized dead-end filtration cells, for the preliminary study of FLD treatment. Figure S2. (a) Cross-flow experimental set-up for investigating FLD treatment with NF membranes, (b) test section. Figure S3. (a) Average permeate flux and (b) nutrient rejection of XLE and NF90 membranes at different operating pressures. Dead-end mode, 1 h of filtration time, Feed: synthetic solutions. Figure S4. Flux temporal variation in tests with NF90 and XLE membranes, in the cross-flow mode. Final permeate recovery ~75 to 76% in both cases. Feed: Synthetic solutions. Figure S5. Effect of permeate recovery on nutrient concentration by (a) NF90 and (b) XLE and rejection by (c) NF90 and (d) XLE membranes. Cross-flow mode. Feed: Synthetic solutions. Figure S6. Flux temporal variation for NF90 and XLE membranes in the cross-flow mode; pressure 5 bar, permeate recovery ~40%, Feed: FLD (sample S1). Figure S7. Results of cross-flow tests with NF90 at 5 bar. (a) Effect of permeate recovery on flux and (b) temporal variation of permeate recovery. Permeate recovery ~45–70%, cross-flow velocity: 20 cm/s. Feed: untreated (1P) and treated (2P-4P) FLD. Figure S8. Variation of normalized flux, versus a) permeate recovery and b) processing time. Data of tests with pretreated FLD feed S5 (#3_9 bar). Figure S9. Effect of permeate recovery on (a) NH_4_-N and (b) PO_4_-P concentration in the retentate (bulk solution); experimental series #3_9 bar. Feed: pretreated FLD (204.1 mg/L ΝH_4_-Ν and 76.0 mg/L ΡO_4_-Ρ). Figure S10. Effect of permeate recovery on nutrients rejection; experimental series #3_9 bar. Feed: pretreated FLD (204.1 mg/L ΝH_4_-Ν, 76.0 mg/L ΡO_4_-Ρ). Figure S11. (a) Dynamic flow chart (HMI), screen. (b) A view of the pilot plant with two pressure vessels in series. Refs. [69,70] are cited in Supplementary Materials.

## Appendix A

**Table A1:** Physicochemical characteristics of the real Filtered Liquid Digestate (FLD) samples, used as feed solution in this study.

	Sample 1 (S1)	Sample 2 (S2)	Sample 3 (S3)	Sample 4 (S4)	Sample 5 (S5)	Limit Values for Reuse [41]	
Months of plant operation	1.5	4	7	8	9.5	Unrestricted irrigation	Restricted irrigation
pH	7.2	7.5	7.1	7.5	7.3	6.5–8.5	
eC, μS/cm	4170	5830	4580	4330	5860	700	3000
Total dissolved solids (TDS), mg/L	2670	3730	2930	2770	3750	450	2000
Total organic carbon (TOC), mg/L	37.6	29.7	19.7	69.6	32	N/S	N/S
COD, mg/L	85	115	65	161	60	N/S	N/S
PO_4_-P, mg/L	134	92.4	85.6	77.7	74	2 *	2 *
NH_4_-N, mg/L	Ν/A	168.4	169.4	173.7	211.7	2 **	N/S
Cl^−^, mg/L	437	515	366	363	431	140	350
SO_4_^2−^, mg/L	72	Ν/A	125.3	-	-	N/S	N/S
Alkalinity, mg CaCO_3_/L	1515	2439	1521	1581	2037	90	500
TSS, mg/L	40	5	0.6	0.2	0.8	10	35

N/A: Not analyzed; N/S: Not specified; * Total Phosphorus; ** Regarding regions characterized as vulnerable to pollution caused by nitrates.

**Table A2:** Physicochemical characteristics of the synthetic feed solutions used in the preliminary study of membrane performance for liquid digestate treatment.

Tested Membrane	XLE						NF90						TS80	ACM2
Operating pressure, bar	3	5	5	5	5	7	3	5	5	5	5	7	5	5
Test duration, min	60	60	453	60	60	60	60	60	463	60	60	60	60	60
pH	4.7	4.7	4.9	5.4	8.5	4.9	4.8	4.9	4.8	5.4	8.5	5.1	4.9	4.9
eC, μS/cm	2060	1854	2270	1539	934	1837	2010	1822	1926	1496	872	1922	1921	1908
Total dissolved solids (TDS), mg/L	1320	1190	1450	985	598	1180	1290	1170	1230	957	558	1230	1230	1220
Osmotic pressure, bar	1.02	0.92	1.12	0.76	0.46	0.91	1.00	0.91	0.95	0.74	0.43	0.95	0.95	0.95
PO_4_-P, mg/L	163	162	129	-	125	146	145	158	168	-	130	148	159	157
NH_4_-N, mg/L	148	147	159	150	-	156	147	152	158	151	-	157	142	150
Alkalinity, mg CaCO_3_/L	ND	ND	ND	ND	ND	ND	ND	ND	ND	ND	ND	ND	ND	ND

ND: Not determined.

**Table A3:** Main physicochemical parameters of feed solutions, conditions, and key process-parameter values of the preliminary tests 1P–4P, performed with real FLD. Cross-flow velocity: 20 cm/s.

Test	0P	1P	2P	3P	4P
Membrane	XLE	NF90	NF90	NF90	NF90
Feed Solution	S1 *	S1 *	S2	S2	S3
Pretreatment	None		Precipitation, separation of the supernatant and addition of acid	Acid addition	Acid addition
pH	7.4		6.6	6.6	6.4
TOC, mg/L	16.5		21.8	15.4	22.9
NH_4_-N, mg/L	104.1		118.6	126.6	151.7
PO_4_-P, mg/L	165.1		11.9	74.8	98
Alkalinity, mg CaCO_3_/L	N/A		1133	1937	1022
eC, μS/cm	3380		4900	4930	3880
Osmotic Pressure, bar	1.67		2.42	2.44	1.92
	Supersaturation index (SI) of constituents having the tendency to precipitate in feed solution				
Aragonite	0.19		−1.62	−0.58	−1.09
Ca_3_(PO_4_)_2_(beta)	2.63		−4.32	−0.28	−0.78
Ca_4_H(PO_4_)3:3H_2_O	1.92		−7.19	−1.55	−2.08
CaHPO4	0.40		−1.76	−0.16	−0.19
CaHPO_4_:2H_2_O	0.12		−2.04	−0.44	−0.47
Calcite	0.37		−1.44	−0.4	−0.91
Dolomite (ordered)	0.51		-	−0.82	−1.7
Hydroxyapatite	10.62		−1.11	5.36	4.4
	Test Conditions				
Applied pressure, bar	5	5	5	5	5
Permeate recovery, %	41.5	40	55.5	53.7	69.5
Initial flux, L/m^2^·h	20	27	22	20	22
Final flux, L/m^2^·h	8	6	6	7	6
Test duration, min	702	600	610	590	975
	Permeate				
pH	8.1	8.0	6.5	6.9	6.6
NH_4_-N, mg/L (Rejection, %)	14.8 (85.8)	8.0 (92.3)	11.5 (94.1)	14.2 (92.4)	21.7 (91.3)
PO_4_-P, mg/L (Rejection, %)	2.3 (98.6)	1.7 (99.0)	0.5 (96.4)	2.2 (98.5)	4.9 (96.5)
SO_4_^2−^	ND	ND	56.3	30.2	75.5
Cl^−^	23.7	46.8	59.1	51.9	94.5
eC, μS/cm (Rejection, %)	277.3 (91.8)	404.7 (88.0)	489.1 (93.3)	341.6 (94.4)	610 (90.5)
	Retentate				
NH_4_-N, mg/L (Cf)	136 (1.3)	155.4 (1.5)	283.2 (2.3)	272.8 (2.2)	480 (3.2)
PO_4_-P, mg/L (Cf)	95 (1.0)	70 (0.9)	24.8 (2.5)	181 (2.4)	193.3 (2.7)
eC, μS/cm	5270	4830	11310	10680	11190
	Supersaturation index of constituents having the tendency to precipitate in retentate solution (recovery 40%)				
Aragonite		1.34	−1.58	−0.38	−1.58
Ca_3_(PO_4_)_2_(beta)		4.71	−2.76	1.97	−2.76
Ca_4_H(PO_4_)_3_:3H_2_O		4.07	−5.13	1.28	−5.13
CaHPO_4_		0.48	−1.25	0.43	−1.25
CaHPO_4_:2H_2_O		0.19	−1.54	0.14	−1.54
Calcite		1.52	−1.4	−0.2	−1.4
Dolomite(disordered)		2.08	-	−1.01	-
Dolomite(ordered)		2.63	-	−0.46	-
Huntite		0.49	-	−5.33	-
Hydroxyapatite		14.71	1.51	9.28	4.4

* Thawed after freezing.

**Table A4:** Main physicochemical parameters of Filtered Liquid Digestate (FLD), pretreated with H_2_SO_4_. Conditions and key process-parameter values of the test series performed for evaluation of the membrane performance in actual conditions. Cross-flow velocity: 20 cm/s, unless otherwise specified.

Series of Experiments	#1			#2 ^a^			#3 ^a^			#4		
Test	1a	1b	1c	2a	2b	2c	3a	3b	3c	4a	4b	4c
Membrane initial condition	FM	DI	CIP	FM	DI	CIP	FM	DI	PERM	FM	DI	CIP
Applied pressure, bar	8				9		9			10		
Clean water permeability, L/m^2^·h·bar ^b^	10.9	6.5	7.1	10.5	7.1	7.1	10.5	6.8	-	9.0	6.3	6.9
Feed Solution	S3			S5			S5			S4		
Pretreatment	Acid addition			Acid addition			Acid addition			Acid addition		
pH	6.7	6.8	6.9	6.5	6.6	6.6	6.6	6.6	6.7	6.5	6.5	6.5
TOC, mg/L	39.2	40.3	38.9	34.7	31.0	29.8	34.8	36.0	34.0	62.7	56.2	46.0
NH_4_-N, mg/L	157.4	164.8	158.4	196.4	202.8	177.1	204.1	194.4	195.4	154.8	157.8	150.3
PO_4_-P, mg/L	74.5	77	74	69.3	69.3	62.5	67.3	62.0	88.6	75.5	74.5	73.0
Alkalinity, mg CaCO_3_/L	1232	1305	1279	1640	1618	1477	1646	1647	1649	1235	1275	1055
eC, μS/cm	4010	4030	3960	4810	4910	4690	4830	4850	4900	3950	4000	3880
TDS, mg/L	2570	2580	2530	3080	3140	3000	3090	3100	3130	2530	2560	2480
Osmotic pressure, bar	1.65	1.71	1.66	1.93	2.12	2.00	2.00	2.05	2.07	1.66	1.61	1.59
	Test Conditions											
Test duration, min	202	167	175	205	162	101	207	178	172	163	144	164
Permeate recovery, %	51.5	41.2	38.7	48.6	37.4	38.2	51.0	39.2	39.4	49.1	38.2	38.4
ΔP_ap_, bar	8	8	8	9	9	9	9	9	9	10	10	10
Initial flux, L/m^2^·h	40	32	33	41	36	36	41	34	35	48	44	42
Final flux, L/m^2^·h	21	24	23	24	26	29	22	23	22	30	28	23
Effective permeability, L/m^2^·h·bar	6.7	5.4	5.7	6.3	5.4	5.7	6.3	5.4	5.5	6.1	5.6	5.6
	Permeate											
pH	6.9	7.0	6.9	7.1	6.8	7.2	6.5	6.5	6.5	6.5	6.6	6.5
eC, μS/cm (Rejection, %)	124.8 (97.6)	128.7 (97.2)	107.7 (97.6)	199.6 (96.8)	165.4 (96.9)	152.8 (97.0)	160.9 (97.4)	156.3 (97.0)	144.7 (97.3)	101.8 (98.2)	88.9 (98.0)	81.5 (98.1)
NH_4_-N, mg/L (Rejection, %)	4.3 (97.8)	5.0 (97.3)	3.7 (97.9)	7.0 (97.3)	7.0 (97.1)	5.8 (87.3)	6.8 (97.5)	6.6 (97.1)	5.5 (97.5)	3.6 (98.1)	3.7 (97.9)	2.9 (98.3)
PO_4_-P, mg/L (Rejection, %)	0.7 (99.3)	0.5 (99.4)	0.4 (99.5)	0.7 (99.2)	0.6 (99.1)	1.5 (97.7)	0.8 (99.1)	0.6 (99.1)	0.7 (99.3)	0.4 (99.6)	0.3 (99.7)	0.2 (99.8)
SO_4_^2−^, mg/L	6.0	5.1	3.7	9.0	7.7	9.8	12.0	6.5	9.7	4.6	3.4	2.8
Cl^−^, mg/L	14.3	12.8	9.8	19.8	16.6	13.4	18.7	16.1	14.5	10.0	6.0	8.0
	Retentate											
NH_4_-N, mg/L Concentration factor, Cf	337.3 C_f_: 2.1	254.2 C_f_: 1.5	248.5 C_f_: 1.6	432.3 C_f_: 2.2	333.5 C_f_: 1.6	289.4 C_f_: 1.6	447.7 C_f_: 2.2	376 C_f_: 1.9	350.6 C_f_: 1.8	297.7 C_f_: 1.9	264.1 C_f_: 1.7	257 C_f_: 1.7
PO_4_-P, mg/L Concentration factor, Cf	160 C_f_: 2.1	123.5 C_f_: 1.6	118 C_f_: 1.5	155.4 C_f_: 2.2	108.1 C_f_: 1.6	115.6 C_f_: 1.8	150.5 C_f_: 2.2	147.0 C_f_: 2.4	147 C_f_: 1.7	149.0 C_f_: 2.0	127.0 C_f_: 1.7	126.0 C_f_: 1.7
eC, μS/cm	8350	6460	6390	10370	8060	7400	10790	8710	8530	7860	6620	6680

FM: Fresh membrane; DI: Flushing with deionized water; CIP: Cleaning protocol; PERM: Flushing with permeate; ^a^ Cross-flow velocity: 22 cm/s; ^b^ Nominal permeability calculated from experimental data provided by the manufacturer: 10.3 L/m^2^·h bar
