# Supplementary material for: Valorization of Anaerobic-Fermentation Liquid Digestates—Membrane-Based Process Development
Source: Membranes (Basel). 2023 Mar 1;13(3):297. doi: 10.3390/membranes13030297 (PMC10051141; doi:10.3390/membranes13030297)
Supplement: Supplementary file 1 [file membranes-13-00297-s001.zip › membranes-2240151-supplementary.pdf]

Supplementary Data

Valorization of anaerobic-fermentation liquid digestates - Membrane-based process development

C. Tsaridou, A. Karanasiou, K. V. Plakas, A. J. Karabelas

Chemical Process and Energy Resources Institute, Centre for Research and Technology-Hellas (CERTH), Thermi-Thessaloniki, 57001 Greece

S1. NF and ULPRO membranes tested

Table S1. Specifications and performance data of the commercial membranes (2540 SWM modules) used in preliminary dead-end filtration tests; footnotes indicate manufacturers' tests conditions [1-4]

Manufacturer	Type	Material	Permeate flow, L/min	Rejection, % NaCl	$MgSO_4$	$CaCl_2$	Na_2SO_4	MWCO, Da	Operation pH	max. Operating Pressure, bar	max. Operating Temperature, °C
**DuPont-**	XLE	TFC, PA	2.2[1]	99[1]	ND	96.9[2]	98.1[2]	<100	2-11	41	45
**Filmtec™**	NF90	TFC, PA	1.8[3]	85-95[3]	>97[4]	98.8[2]	99.9[2]	200	3-10	41	45
**Microdyn-**	TS80	TFC	1.6[5]	80[6]	98.5[5]	ND	ND	ND	1-12	41	45
**Nadir GMBH**	ACM2	TFC	1.7[7]	>99[7]	ND	ND	ND	ND	1-12	41	45

TFC: Thin-film composite; PA: Polyamide; [1] 500 mg/L NaCl, 6.9 bar, 25 °C, 15% permeate recovery; [2] 1170 mg/L $CaCl_2/Na_2SO_4$, 5 bar, 25 °C, pH= 6-7; [3] 2000 mg/L NaCl, 4.8 bar, 25 °C, 15% permeate recovery; [4]2000 mg/L $MgSO_4$, 4.8 bar, 25 °C, 15% permeate recovery; [5] 2000 mg/L $MgSO_4$, 7.6 bar, 25 °C, 15% permeate recovery, pH 8.0, 30 min operation; [6] 2000 mg/L NaCl, 7.6 bar, 25 °C, 15% permeate recovery, pH 8.0, 30 min; operation; [7] 2000 mg/L NaCl, 15.5 bar, 25 °C, 15% permeate recovery, pH 8.0, 30 min operation

S2. Experimental set-ups and analytical equipment

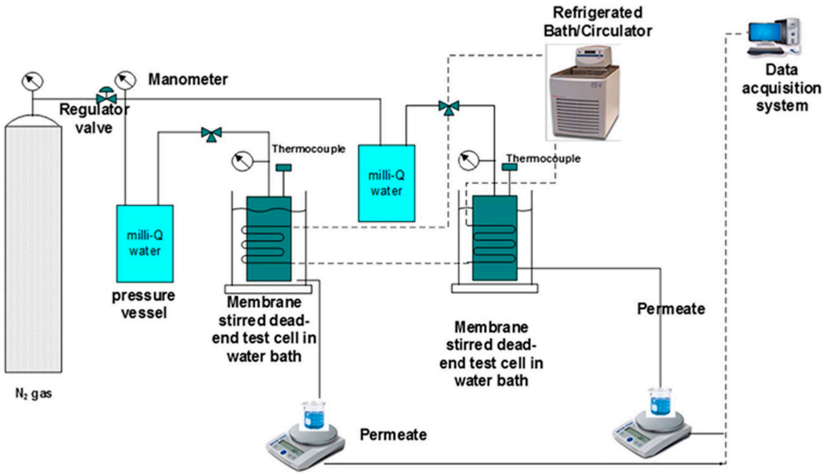

Figure S1. Experimental set-up, involving pressurized *dead-end filtration cells*, for the preliminary study of FLD treatment [5].

Table S2. Instruments used for characterization of membrane performance in the cross-flow filtration tests

Parameter	Instrument / Method
**pH**	Bench meter AG 744 Metrohm, Switzerland
**Electric Conductivity, eC**	Portable multimeter Multi 3510 IDS, WTW, Germany
**TOC**	TOC-L Analyzer, Shimadzu, Japan
**Ions**	Ion Chromatography (Prominence, Shimadzu, Japan)<br>Cations: Column IC SI-52 4E (Shodex, Japan) and methanesulfonic acid 4.0 mM as mobile phase<br>Anions: Column IC YS-50 (Shodex, Japan) and sodium carbonate 3.6 mM (anions) as mobile phase
**Alkalinity**	Titrator 877 Titrino Plus, Metrohm AG, Switzerland
**Membrane surface observations**	SEM JSM-IT500LV, Jeol, Japan
**Membrane surface composition**	EDS X-ACT, Oxford, UK

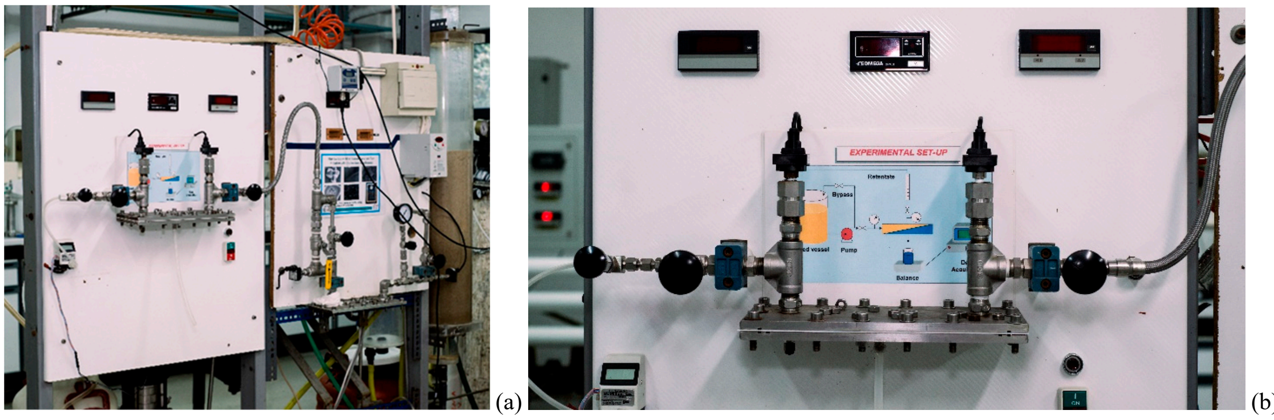

(a) (b)

Figure S2. (a) Cross-flow experimental set-up for investigating FLD treatment with NF and LPRO membranes, (b) test section

S3. Experimental results with synthetic solutions

Table S3. Comparison of nutrients rejection and permeate flux, using single-nutrient (NH_4-N or P-PO_4) and 'binary' (NH_4-N plus P-PO_4) synthetic feed solutions, in dead-end filtration mode; applied pressure 5 bar.

	**"Binary" Solution**	**NH_4-N Solution**	**P-PO_4 Solution**
		**XLE**	
**Flux, L/m²h**	32.6	38.2	31.4
**NH_4-N rejection, %**	84	84	-
**PO_4-P rejection, %**	99	-	95
		**NF90**	
**Flux, L/m²h**	29.7	45.4	40.3
**NH_4-N rejection, %**	89	75	-
**PO_4-P rejection, %**	98	-	97

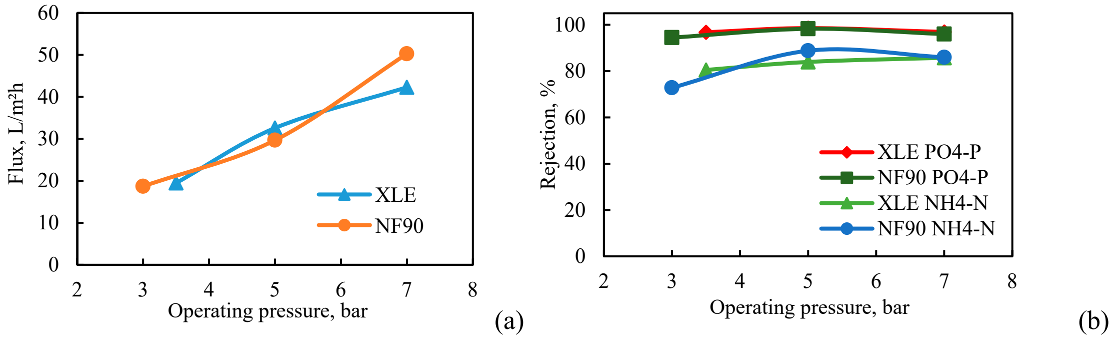

Figure S3. (a) Average permeate flux and (b) nutrients rejection of XLE and NF90 membranes at different operating pressures. *Dead-end mode*, 1 hour of filtration time, Feed: synthetic solutions.

Table S4. Results of the rejection tests performed in the *cross-flow* set-up with XLE and NF90 membranes. Feed: Synthetic solutions, applied pressure ~5 bar, ~25 ºC, cross-flow velocity ~20 cm/s.

Parameter	XLE	NF90
**Initial flux, L/m²h**	30	~43
**Initial permeability, L/(m²·h·bar)**	7.9	12.3
**Test duration, min**	480	330
**Permeate recovery, %**	75	76
**Feed solution:**		
**NH_4-N, mg/L**	165	173
**PO_4-P, mg/L**	165	129
**Retentate:**		
**NH_4-N, mg/L**	471	381
**PO_4-P, mg/L**	600	537
**Concentration factor, C_f:**		
**NH_4-N**	2	2.2
**PO_4-P**	3.1	4.2
**Rejection, %:**		
**NH_4-N**	83.5	83
**PO_4-P**	98.5	97.1

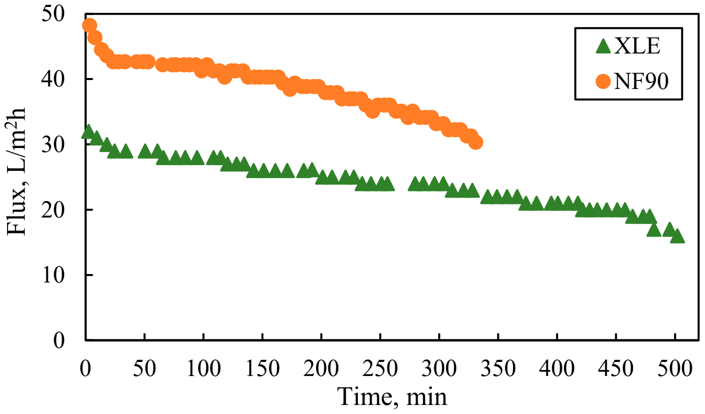

Figure S4. Flux temporal variation in tests with NF90 and XLE membranes, in the *cross-flow mode*. Final permeate recovery ~75 to 76% in both cases. Feed: Synthetic solutions.

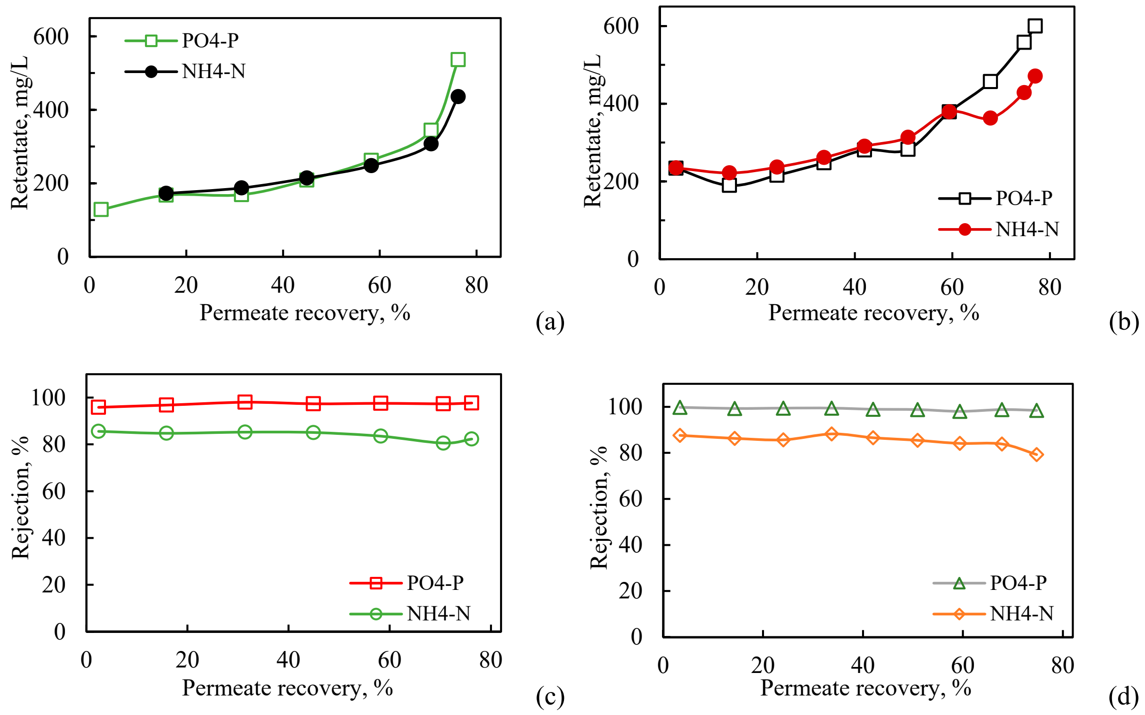

Figure S5. Effect of permeate recovery on nutrients concentration by (a) NF90 and (b) XLE and rejection by (c) NF90 and (d) XLE membranes. *Cross-flow mode*. Feed: Synthetic solutions

S4. Experimental results with FLD

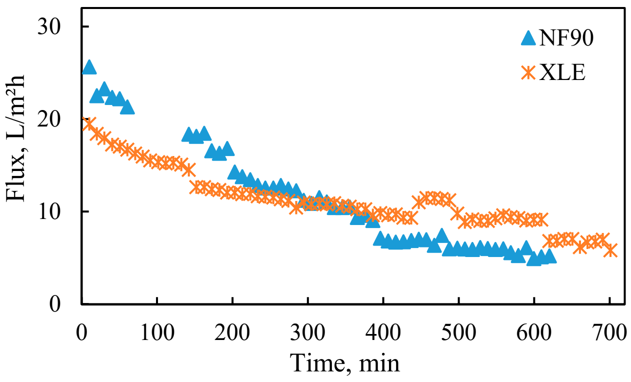

Figure S6. Flux temporal variation for NF90 and XLE membranes in the *cross-flow mode*; pressure 5 bar, permeate recovery ~40%, Feed: FLD (sample S1).

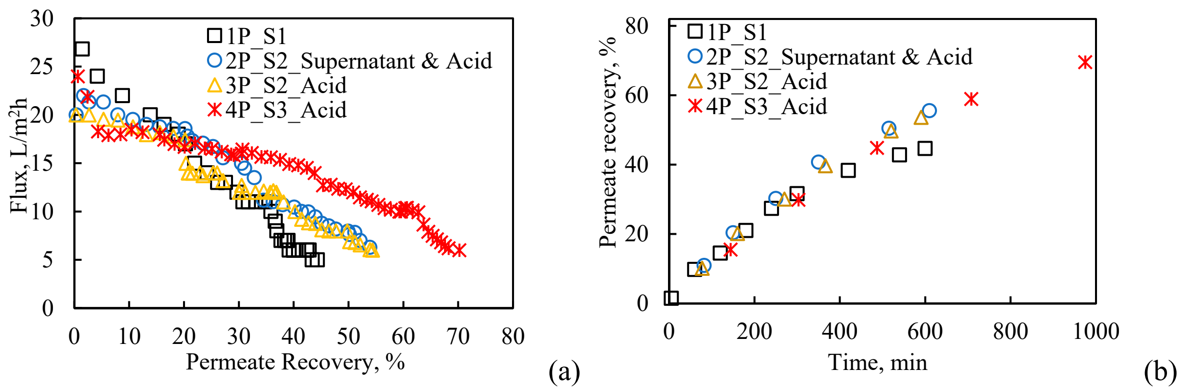

Figure S7. Results of *cross-flow* tests with NF90 at 5 bar. (a) Effect of permeate recovery on flux and (b) temporal variation of permeate recovery. Permeate recovery ~45-70%, cross flow velocity: 20 cm/s. Feed: untreated (1P) and treated (2P-4P) FLD.

Table S5. Foulant species mass-surface density on NF90 membrane (preliminary tests 1R-3R at 5 bar). Dissolution technique employed, involving HCl 60mM and NaOH 25mM; average values from both test-sections (1 and 2).

	**Untreated FLD sample**	**Pretreated FLD sample**	
**Test**	**1P***	**2P**	**3P**
	**Estimated foulant species surface density on NF90 membrane, mg/m^2**		
**P**	943	ND	ND
**Ca**	4,169 for section 1<br>2,316 for section 2	12	13
**Mg**	329	2	3.9
**K**	398	ND	ND
**Na**	2,782	194	232
**Zn**	ND	0.9	0.8
**Ni**	ND	2.2	2.8
**Fe**	ND	2.0	2.2
**TOC**	63	86	87

ND: Not Determined; *Dissolution test only with HCl solution

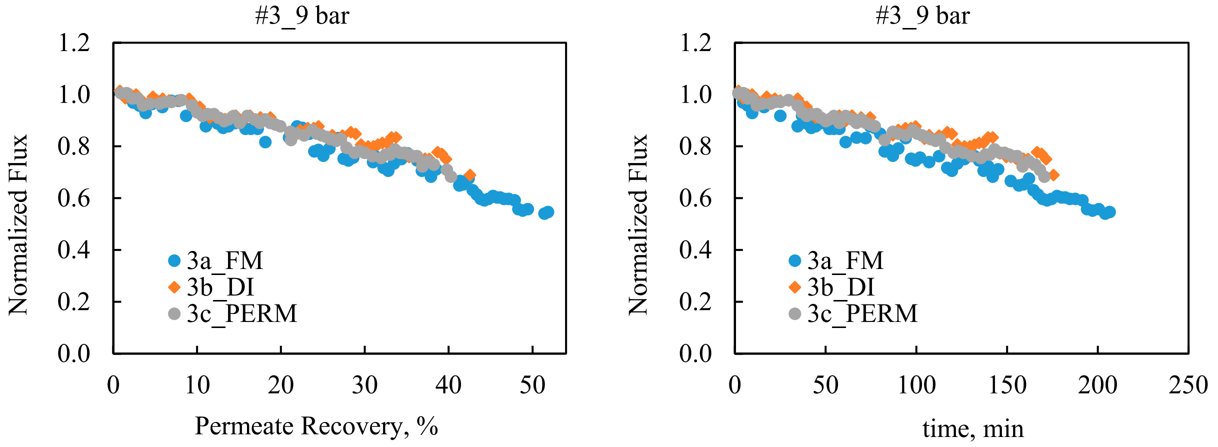

Figure S8. Variation of normalized flux, versus a) permeate recovery and b) processing time. Data of tests with pretreated FLD feed S5 (#3_9 bar).

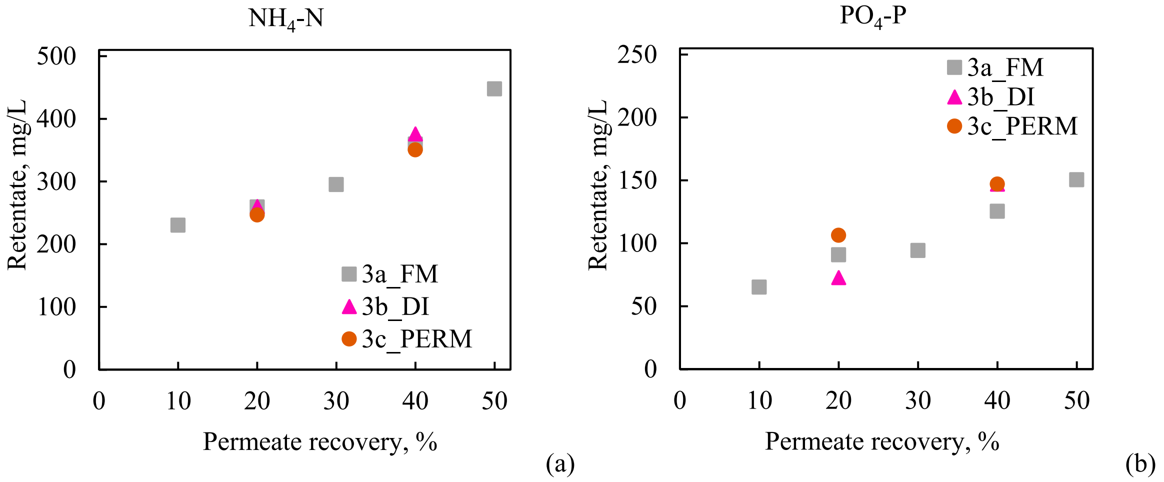

Figure S9. Effect of permeate recovery on (a) NH_4-N and (b) PO_4-P concentration in the retentate (bulk solution); experimental series #3_9 bar. Feed: pretreated FLD (204.1 mg/L NH_4-N and 76.0 mg/L PO_4-P).

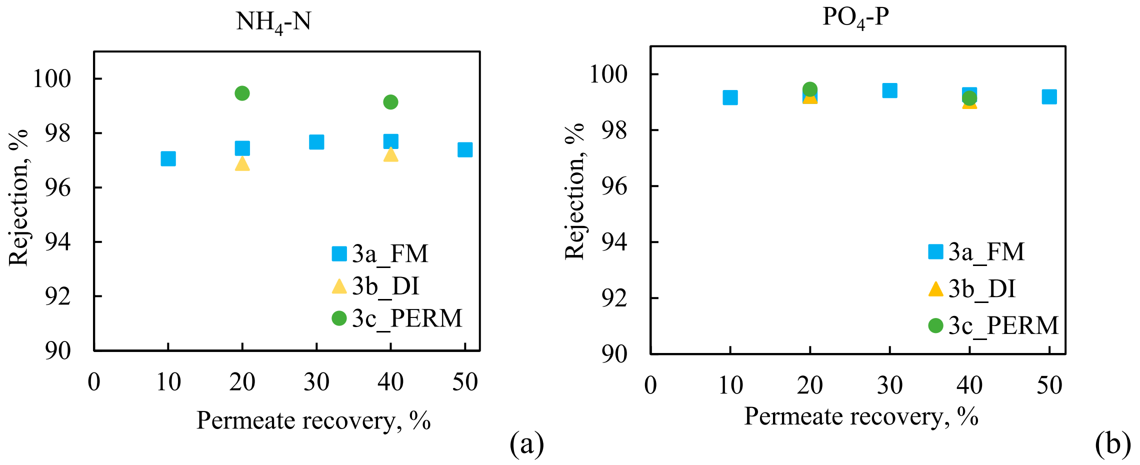

Figure S10. Effect of permeate recovery on nutrients rejection; experimental series #3_9 bar. Feed: pretreated FLD (204.1 mg/L NH_4-N and 76.0 mg/L PO_4-P).

S4. Process simulation parameters

Table S6. Input data for the Simulators WAVE (Dupont) and NRRE/SWM, corresponding to S3 feed-solution characteristics

**Simulator**	**WAVE (DuPont)**	**Simulator**	**NRRE/SWM**
**Type of solution:**	Wastewater	**Temperature, °C:**	25
**Water type:**	With conventional pretreatment	**Feed solution salinity, kg/m³ *:**	2.93
**Silt Density Index (SDI):**	<5	**Density, kg/m³:**	999.283
**Temperature, °C:**	25	**Viscosity, kg/(m·s):**	0.0008945
**TDS, mg/l*:**	3067	**Diffusivity, m²/s:**	1.3 $*10^{-9}$ [5]
**TSS, mg/l:**	0.6	**Osmotic coefficient, Pa·m³/kg:**	77474
**Organics (TOC), mg/l:**	19.6	**Permeate recovery, %:**	50
**Turbidity, NTU:**	18.1	**Inlet velocity (u), m/s:**	0.2
**pH:**	7.0	**Membrane resistance, m⁻¹:**	$5.7 \cdot 10^{13}$ (Permeability=7.1 L/m²h·bar)
**Number of Elements:**	4 (NF90 2540)	**Number of elements:**	4 (2540 with 28 mil feed spacer)
**Permeate Recovery, %**	50	**Permeate Recovery, %**	50

*The slight difference in TDS concentration of the feed solution of WAVE (3,069 mg/L) compared to that of NRRE/SWM Simulator (2,930 mg/L), is likely due to the fact that in the case of WAVE, the feed water composition (i.e. the TDS concentration) is computed automatically, via the summation of the input ions concentration and cannot be entered by the user.

Table S7. WAVE Simulator operation limits (design warnings) for the input data of Table S6.

**Design Warning**	**Limit**	**Value**	**Element**
Permeate flow rate > Maximum limit (L/min)	1	2.3	1
Permeate flow rate > Maximum limit (L/min)	1	2.0	2
Permeate flow rate > Maximum limit (L/min)	1	1.8	3
Permeate flow rate > Maximum limit (L/min)	1	1.5	4
Element recovery > Maximum limit (%)	12	15.2	1
Element recovery > Maximum limit (%)	12	15.8	2
Element recovery > Maximum limit (%)	12	16.3	3
Element recovery > Maximum limit (%)	12	16.5	4

Table S8. Input data for a small/middle scale pilot plant simulation with NRRE/SWM Simulator (test series #3_9 bar with pretreated FLD).

**Input parameter**	
**Temperature, °C**:	25
**Feed solution salinity, kg/m^3**:	3.1
**Density, kg/m^3**:	999.390
**Viscosity, kg/m/s**:	0.0008949
**Diffusivity, m^2/s**:	$1.3 \cdot 10^{-9}$ [6]
**Osmotic coefficient, Pa·m^3/kg**:	77432
**Inlet velocity** (u), **m/s**:	0.22
**Membrane resistance, m^{-1}**:	$7.49 \cdot 10^{13}$ (Permeability=5.4 L/m^{2}h·bar)
**Number of elements**:	2; Permeate recovery 20%
	4; Permeate recovery 40-44%

Table S9. Simulations of a small/middle scale pilot plant with NRRE/SWM simulator (input data from Table S8).

	**Input data**		**Output data**		
**Simulation case No**	**No of membrane modules in series**	**Permeate Recovery, %**	**Inlet Pressure, bar**	**Feed Flow, L/min**	**Flux, L/m^{2}h***
**1**	2	21.0	9.5	13.5	32.0
**2**	4	39.5	9.0	13.3	32.0
**3**	4	40.9	9.2	13.3	32.8
**4**	4	44.4	10.2	13.3	37.5

* At the exit of first in series membrane module

S5. Protocol of experimental series #1 to #4 - Cleaning Procedures

1. Clean (deionized) water test with new/fresh NF90 coupons and determination of membrane clean water permeability. Applied pressure: 8, 9, 10 bar.
2. First rejection test (#Xa_FM) with acidified FLD (S3-S5) for permeate recovery up to ~50% and collection of samples at time intervals corresponding to 10% recovery. Applied pressure: 8, 9, 10 bar.
3. Flushing the system with DI water at 0,68 L/min until eC reduction to < 40 μS/cm. Second clean water test (40-60 min) for determination of membrane clean water permeability. Applied pressure: 8, 9, 10 bar.
4. Second rejection test (#Xb_DI) with the acidified FLD (S3-S5) for permeate recovery up to ~40% and collection of samples at time intervals corresponding to 20% recovery. Applied pressure: 8, 9, 10 bar.
5. Chemical cleaning (CIP - Clean In Place) of system with sodium hydroxide (NaOH) 0.1% w/w for alkaline cleaning (pH 12), followed by hydrochl. acid (HCl) 0.2% w/w for acidic cleaning (pH 1-2), according to manufacturer's instructions [7]:
   a. Recycling of cleaning solution at low flow rate (0,33 L/min) without permeation for 10-15 min.
   b. Soaking for 10-15 min
   c. Recycling of cleaning solution at high flow rate (1,00 L/min) without permeation for 30 min
   d. Flushing at 1,00 L/min without permeation until reduction of conductivity to < 40 μS/cm and clean water testing for 40 to 60 min

   In the test series #3_9 bar, the CIP was replaced by flushing the system with the produced permeate (PERM).
6. Third clean water test. The duration of clean water tests was 40-60 min. Applied pressure: 8, 9, 10 bar.
7. Third rejection test (#Xc_CIP/PERM) with acidified FLD (S3-S5) for permeate recovery up to ~40% and collection of samples at time intervals corresponding to 20% recovery. The same pressure was applied in all rejection tests #Xa-c of each experimental series. Applied pressure: 8, 9, 10 bar.

8. Removal of the membrane specimen from the first in row test-section for further analysis; i.e. SEM/EDS, dissolution and determination of organic and inorganic deposits on the membrane.
9. Flushing the system with DI water and removal of membrane specimen from the second test-section for further analysis.

S6 Pilot plant – Preliminary tests

The pilot plant, depicted in **Figure S11**, was designed and constructed in the Natural Resources and Renewable Energies Laboratory, Chemical Process and Energy Resources Institute at CERTH. Views are provided of the control touch-screen (Fig. 10a) and of the main pilot unit (Fig.10b).

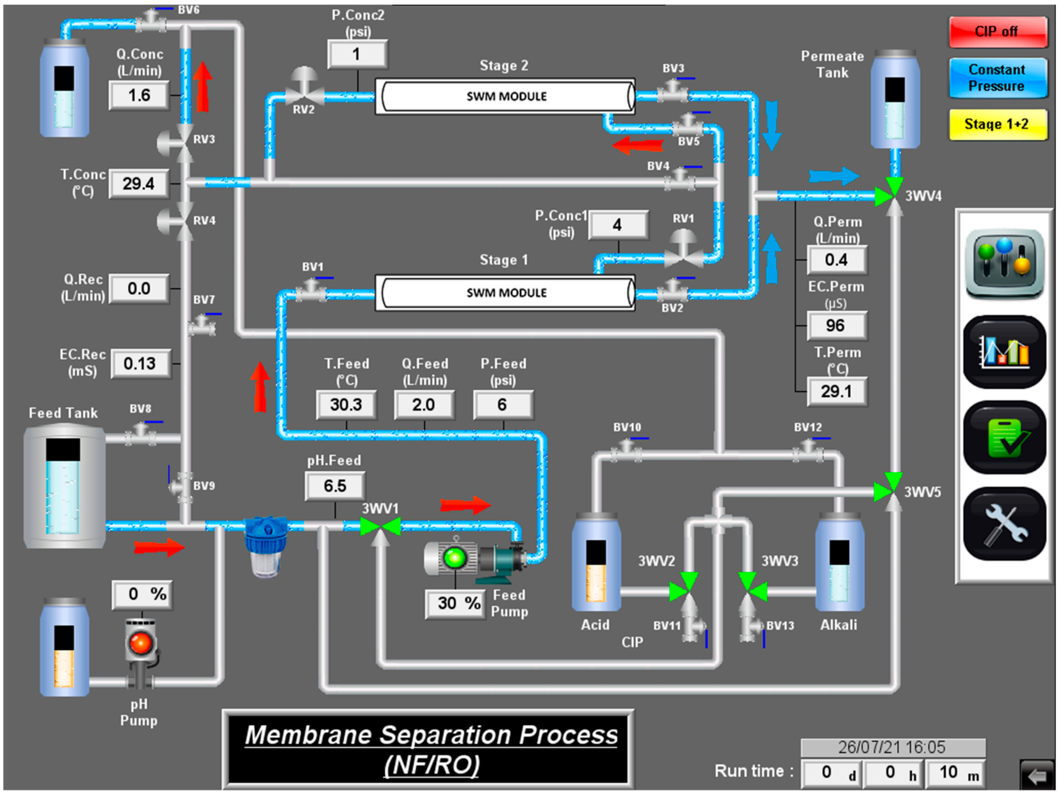

(a)

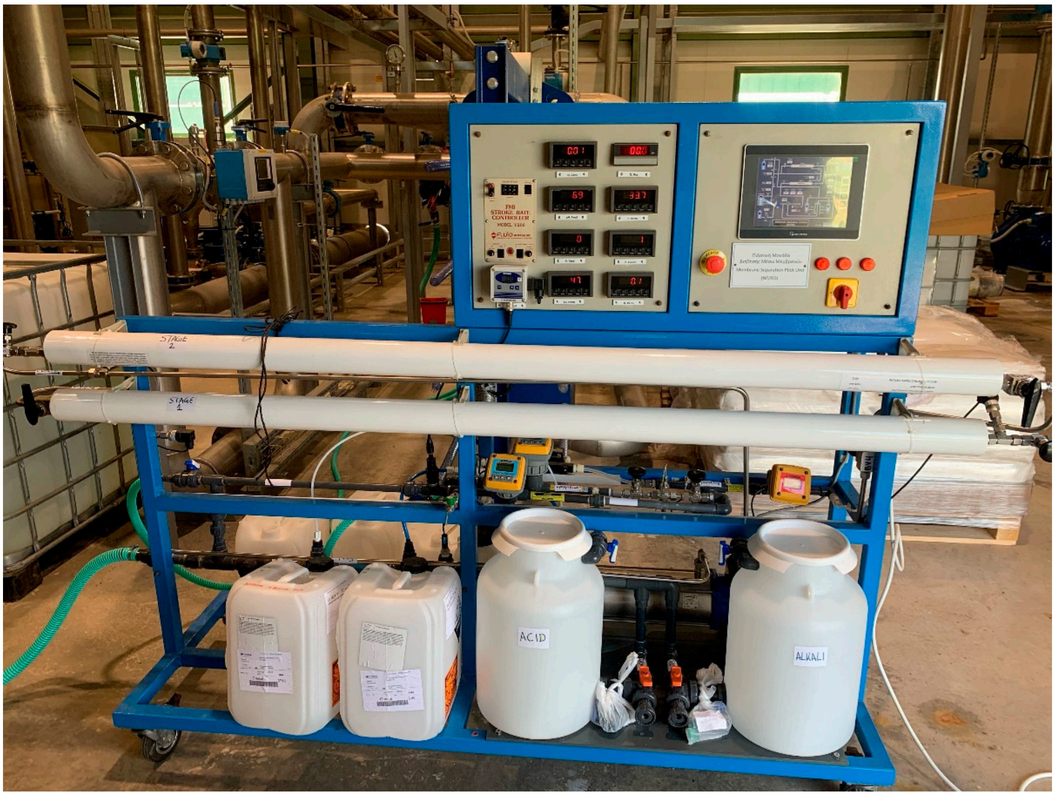

(b)

Figure S11. (a) Dynamic flow chart (HMI), screen. (b) A view of the pilot plant with two pressure vessels in series

Table S10. Composition of synthetic feed-solutions and rejection results of pilot tests.

	Na^+, mg/L	Ca^{2+}, mg/L	Cl^-, mg/L	SO_4^{2-}, mg/L	eC, μS/cm*
**Feed tank**	1,344 ± 25	148 ± 25	327 ± 33	2,038 ± 123	4,561 ± 67
		**1 Stage (2 SWM modules)**			
**Concentrate**	1,631 ± 61	195 ± 9	375 ± 5	2,477 ± 38	5,632 ± 34
**Permeate**	17.5 ± 0.1	0.34 ± 0.05	15.3 ± 0.1	8.8 ± 0.1	99.4 ± 0.7
**Rejection, %**	98.7	99.8	95.3	99.6	97.8
		**2 Stages (4 SWM modules)**			
**Concentrate**	1,812 ± 16	218 ± 2	420 ± 13	2807 ± 3	6,140 ± 77
**Permeate**	35.8 ± 0.5	0.34 ± 0.02	28.0 ± 1.4	10.4 ± 0.6	152.7 ± 4.9
**Rejection, %**	97.3	99.8	91.4	99.5	96.7

* Pilot-unit sensor data

Table S11. Experimental conditions and input data for pilot test with synthetic solution under constant pressure

**Input parameter**	**Values**
**Temperature, °C**	30.7
**Density, kg/m^3**	997.6
**Viscosity, kg/m/s**	0.0007903
**Diffusivity, m^2/s**	$1.3 \cdot 10^{-9}$ [6]
**Osmotic coefficient, Pa·m^3/kg**	74295
**Inlet* flow rate, L/min**	13.0

**Inlet* cross-flow velocity (u), m/s**	0.21
**Membrane permeability, L/m^{2}h·bar**	10.3**
**Membrane resistance, m^{-1}**	4.42· 10^{13}
**1st Element inlet pressure, kPa**	570 (5.7 bar)

* At the entry of 1st SWM module

** Manufacturer data for clean membrane. Non-fouling conditions

References

1. DuPont™, XLE Product Data Sheet. 2021. Available online: https://www.dupont.com/content/dam/dupont/amer/us/en/water -solutions/public/documents/en/RO-FilmTec-XLE-Commercial-PDS-45-D01515-en.pdf (accessed on 8 May 2021).
2. DuPont™, NF90 Product Data Sheet. 2021. Available online: https://www.dupont.com/content/dam/dupont/amer/us/en/water -solutions/public/documents/en/NF-FilmTec-NF90-Commercial-Systems-PDS-45-D01520en.pdf (accessed on 8 June 2021).
3. Microdyn-Nadir, Product Specification - TRISEP® TS80. 2021. Available online: https://www.microdyn-nadir.com/wp-content/uploads/TS80.pdf (accessed on 8 June 2021).
4. Microdyn-Nadir, Product Specification - TRISEP® ACM2. 2021. Available online: https://www.microdyn-nadir.com/wp-content/uploads/ACM2.pdf (accessed on 8 July 2021).
5. Karabelas, A.J.; Karanasiou, A.; Sioutopoulos, D.C. Experimental study on the effect of polysaccharides on incipient membrane scaling during desalination. *Desalination* **2017**, *416*, 106-121.
6. van Voorthuizen, E.M.; Zwijnenburg, A.; Wessling, M. Nutrient removal by NF and RO membranes in a decentralized sanitation system. *Water Research* **2005**, *39*, 3657-3667.
7. DuPont™, Cleaning Procedures for FilmTec™ FT30 Elements, 2020. Available online: https://www.dupont.com/content/dam/dupont/amer/us/en/water-solutions/public/documents /en/RO-NF-FilmTec-Cleaning-Procedures-Manual-Exc-45-D01696-en.pdf (accessed on 9 November 2021).
